# Photobleaching in STED nanoscopy and its dependence on the photon flux applied for reversible silencing of the fluorophore

**DOI:** 10.1038/s41598-017-09902-x

**Published:** 2017-09-12

**Authors:** Joanna Oracz, Volker Westphal, Czesław Radzewicz, Steffen J. Sahl, Stefan W. Hell

**Affiliations:** 1 Max Planck Institute for Biophysical Chemistry, Department of NanoBiophotonics, Am Fassberg 11, 37077 Göttingen, Germany; 2 University of Warsaw, Faculty of Physics, Pastera 5, 02-093 Warsaw, Poland; 3 Max Planck Institute for Medical Research, Department of Optical Nanoscopy, Jahnstr. 29, 69120 Heidelberg, Germany

## Abstract

In STED (stimulated emission depletion) nanoscopy, the resolution and signal are limited by the fluorophore de-excitation efficiency and photobleaching. Here, we investigated their dependence on the pulse duration and power of the applied STED light for the popular 750 nm wavelength. In experiments with red- and orange-emitting dyes, the pulse duration was varied from the sub-picosecond range up to continuous-wave conditions, with average powers up to 200 mW at 80 MHz repetition rate, i.e. peak powers up to 1 kW and pulse energies up to 2.5 nJ. We demonstrate the dependence of bleaching on pulse duration, which dictates the optimal parameters of how to deliver the photons required for transient fluorophore silencing. Measurements with the dye ATTO647N reveal that the bleaching of excited molecules scales with peak power with a single effective order ~1.4. This motivates peak power reduction while maintaining the number of STED-light photons, in line with the superior resolution commonly achieved for nanosecond STED pulses. Other dyes (ATTO590, STAR580, STAR635P) exhibit two distinctive bleaching regimes for constant pulse energy, one with strong dependence on peak power, one nearly independent. We interpret the results within a photobleaching model that guides quantitative predictions of resolution and bleaching.

## Introduction

Stimulated emission depletion (STED) nanoscopy^1^ is now a well-established method which allows minimally invasive optical access to length scales of tens of nanometers, in fixed and living biological specimens^2^. In addition to the focused excitation beam of confocal laser scanning microscopy, a now widespread implementation of STED uses a doughnut-shaped de-excitation profile to keep fluorophore molecules outside the central nanoscale region effectively in the ground state for the duration of detection at a given scan coordinate. The STED light’s role is therefore to transiently keep molecules non-fluorescent and briefly switch fluorescence “off”. Two key strengths of STED nanoscopy in comparison to other super-resolution fluorescence methods are the universality of its resolution-enabling fluorescence off-switching mechanism, allowing STED to operate well with many different markers, and its ability to image fast dynamics at video rate and beyond^3–5^. The presence of sufficiently intense STED light guarantees the off-switching of fluorophores by stimulated emission, except at the central intensity minimum (ideally, a zero of intensity) of the doughnut, where the “on”-state is established. The spatial resolution in STED nanoscopy is consequently no longer limited by the extent of the focal intensity distribution set by diffraction, but depends on the creation of the “on”-“off” state difference among fluorophores by switching.

To record a super-resolved image by scanning, the fluorescence ability of fluorophores at positions near the targeted coordinate (region of the doughnut) has to be switched off many times anew, with typical peak intensities present in the off-switching profile on the order of ~1 GW/cm^2^ (ref. 6). This high irradiance at the doughnut crest poses challenges, notably the photobleaching of fluorescent markers, in particular of the widely employed small-molecule organic dyes. Several chemical approaches have been demonstrated to partially mitigate this problem, including the development of new fluorescent dyes^7, 8^, decreasing the concentration of free oxygen to minimize destructive chemical reactions^9^, quenching of the triplet state^10^, as well as different optical strategies, such as: use of longer STED pulses to avoid multiphoton excitation^11^, fast scanners or lasers with low repetition rates to allow triplet state relaxation between two successive pulses^12–14^, or other approaches which minimize unnecessary exposure to the intense light^15–17^.

Despite the success of STED methods with different implementations and laser schemes – from pulsed Ti:sapphire lasers to continuous wave (CW) diode lasers and, more recently, nanosecond pulsed fiber lasers – the full potential of the STED concept has not been realized. While <10 nm resolution has been achieved for ultrastable fluorescent color centers in diamond^18, 19^, closing the gap to molecular resolution for small organic fluorophores that are subject to bleaching remains a challenge. The question which experimental parameters hold the key to achieving routine STED resolution levels well below the present 20–30 nm mark remains unanswered. One main reason for this is that it has been difficult to compare different, disparate experiments and implementations of STED, and conclude on the importance of individual parameters. In general, simultaneous determination of photobleaching impact and the resolution achieved is challenged by the fact that higher resolution is typically associated with more pronounced photobleaching. This makes repetitive measurements to gain the necessary statistics on both quantities difficult to acquire. The situation is even more complicated in biological specimens, which often feature variable local environments and randomly varying fluorophore distributions. Moreover, the non-uniform intensity of the doughnut-shaped STED profile in the focal plane causes a spatial dependence of the STED-beam-induced photobleaching. This spatial variability of bleaching is integrated over in the signal collection, thus limiting definitive conclusions.

Here, we report a detailed study of molecular photobleaching and de-excitation efficiency in STED nanoscopy with organic fluorophores. The contribution to signal related to the unwanted absorption of photons from the STED beam (STED-light-induced fluorescence) is also systematically assessed. We investigated how the intensity impacts bleaching over a large range of pulse peak powers and energies. Our study was enabled by thorough experimental design to reduce the variability of conditions and error of measurement. Concretely, to obtain quantitative results on the impact of bleaching as a function of the flux of STED-light photons, it was necessary to perform measurements with a nearly uniform spatial light distribution in the focal plane, and in a bright sample to acquire the necessary statistics in a reasonable measurement time. We also had to assure a relatively uniform, stable local environment and avoid the fluorescence quenching often observed for densely labelled biological samples^20^ or fluorescent beads, as well as the heterogeneity of the single-molecule approach to photobleaching measurements^21^. Measurements with overlapped Gaussian excitation and de-excitation focal spots inside a non-concentrated dye solution fulfilled these requirements and allowed to obtain quantitative data for important parameters in STED microscopy, at the popular wavelength of 750 nm applied here: fluorophore de-excitation (i.e., reversible fluorescence silencing by excited-state depletion), irreversible photobleaching, and excitation by absorption of STED-light photons (resulting in STED-light-induced fluorescence) for various STED photon fluxes. Measurements were performed for several commercially available dyes using a fluorescence recovery approach^22, 23^. We modified the pulse duration of STED by spectral phase shaping of the Ti:Sapphire laser emission by either a spatial light modulator (SLM) for the short pulse regime, or different lengths of optical fibers for the long pulse regime. In the first case, we were able to create arbitrarily shaped STED pulses of durations up to a few picoseconds (further increases of the pulse duration were limited by the maximal phase retardation of the SLM and the finite laser bandwidth). Using the second approach, we controlled the pulse durations up to ~500 ps. We compared results from experiments across this large range of pulse durations to CW and gated CW (gCW) STED operation^24–26^. Further, we examined the experimental data within a simple photobleaching model, which predicts the influence of STED photon distributions in imaging. The model allowed us to obtain information that is difficult to access experimentally, namely the resolution and spatially dependent probability of photobleaching with respect to the targeted coordinate. The goal was to identify optimal parameters for providing the STED de-excitation photons (in the time domain), previously studied theoretically^27^ without including photobleaching, to collect maximal information from the sample during imaging, while at the same time reducing undesirable interactions of fluorophores with the photons applied, conceptually, for de-excitation only. When working with the model to guide choices of future experimental parameters, we were interested in temporal photon distributions within the natural fluorescence decay of the fluorophores studied. CW de-excitation was not considered in our theoretical modelling. This modality, while it features technical simplicity and lower cost of implementation, has the drawback that a constant photon flux acts on the sample also at times when all the fluorophores are already in the dark “off”-state (no fluorescence is expected), which results in increased STED-light-induced fluorescence and bleaching during these times.

## Methods

### Experimental setup

**Figure 1:**
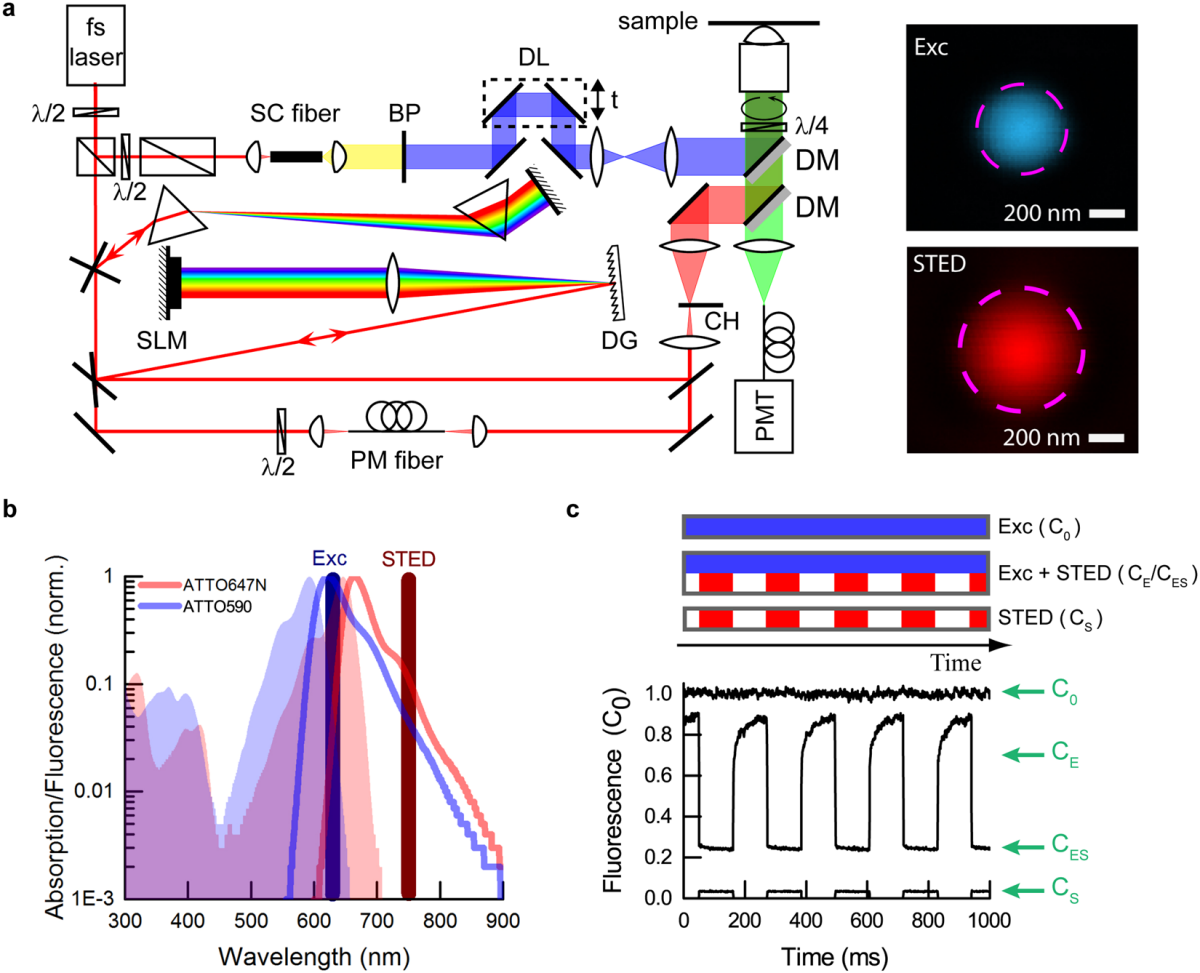
Experimental setup and measurement principle to extract STED de-excitation, bleaching and STED-light-induced fluorescence. (**a**) Microscope with controllable fluxes of STED de-excitation photons. The laser was a femtosecond oscillator operating at 750 nm. The excitation pulse was generated in a supercontinuum (SC) fiber. The excitation wavelength was spectrally selected by the bandpass filter BP (635 nm/10 nm) and synchronized to the de-excitation pulse by the optical delay line DL. The STED pulse shape was controlled in two ways: for short pulses (0.1–3 ps), the optical path consisted of SF11 prisms as pre-compressor and a home-built pulse shaper. The pulse shaper was formed by the diffraction grating DG, the long focal-length lens and the spatial light modulator SLM placed in the Fourier plane created by those elements. For long pulses (10–500 ps, CW), the optical path consisted of polarization-maintaining (PM) fibers of various lengths. CW operation was obtained by preventing mode-locking of the Ti:sapphire laser, and an additional diode laser was used as excitation source in this case (path not shown). To register fluorescence recovery curves, the STED beam intensity was chopped. The excitation (blue) and de-excitation (red) pulses were coupled into the microscope by dichroic mirrors DM and focused into the sample by an objective lens. The fluorescence (green) was collected in back-propagation and registered by a hybrid photomultiplier PMT in a confocal arrangement, with the data acquisition synchronized to the chopper wheel (CH). The insets represent the focal spots of excitation (Exc) and STED beam, as measured by scattered signal of 80 nm gold beads. The dashed circles correspond to the FWHM of intensity. (**b**) Normalized spectral properties of Atto647N and Atto590, with the respective laser wavelengths for excitation and STED. The detection was centred at 690 nm and had 60 nm width (not shown). (**c**) Example excerpt of raw data set for a single measurement (Atto647N), with characteristic levels extracted to derive bleaching, de-excitation and STED-light-induced fluorescence. Each measurement consisted of three intensity traces (curves), registered sequentially: excitation-only, excitation with chopped STED and chopped STED only (acquisition time in each case: ~100 s). C_0_, C_E_, C_ES_, C_S_ denote respectively: C_0_ the initial signal of fluorescence (due to the excitation beam), C_E_ the fluorescence signal right after end of exposure to the STED beam, C_ES_ the residual fluorescence signal in the presence of both the excitation and STED (de-excitation) beam, and C_S_ the fluorescence signal caused by the STED light. Signals correspond to respective fractions of fluorophores. Excitation: ~500 fs pulse duration (FWHM), 30 μW average power; STED (shown measurement): ~25 ps pulse duration (FWHM), 50 mW average power. The delay between excitation pulse and STED pulse was ~50 ps. All powers were measured at the back aperture of the objective lens. The powers in the focal plane were ~70% of these values due to cut-off at the entrance pupil and transmission of the objective lens.

The experimental setup is presented in Fig. 1a. Both the excitation and the STED pulse were created using a commercial mode-locked Ti:Sapphire laser (MaiTai HP, Spectra-Physics) operating at 750 nm wavelength with 80 MHz repetition rate and initial pulse duration of ~100 fs (8.5 nm bandwidth). The excitation pulse (shown in blue), with duration of a few hundred femtoseconds, was generated in a supercontinuum fiber (SCG-800, Newport) and limited to 10 nm bandwidth at 635 nm central wavelength by a laser line clean-up filter BP (Z635/10X, Chroma). The STED pulse (shown in red) was temporally shaped in different ways, depending on the required pulse duration. For short pulses (0.1–3 ps) a home-built prism pre-compressor and pulse shaper were used. The compressor comprised two SF11 prisms which pre-compensate the later optical elements. The pulse shaper consisted of a diffraction grating DG (2400 lines/mm, PC 2400 NIR, Spectrogon), a long focal length lens f = 400 mm and a liquid crystal spatial light modulator SLM (SLM-S640d, Jenoptik) in double-pass configuration. Temporal shaping was performed by applying different spectral phases to the de-excitation pulse. For long pulses (>10 ps), polarization-maintaining (PM) fibers of different lengths were used (PM630-HP, Thorlabs; PMC-600-4, Schäfter&Kirchhoff; PMJ-A3AHPC, OZ Optics). The relative time between excitation and STED pulses was adjusted by an optical delay line DL (Supplementary Information). CW measurements were obtained by suppressing the mode-locking of the Ti:Sapphire laser and using an additional pulsed excitation laser at 80 MHz (LDH-P-635, PicoQuant). All beams were expanded and coupled into the microscope using dichroic mirrors DM. The objective was an immersion oil lens of high numerical aperture (HCX PL APO 100×/1.4NA, Leica). The excitation and STED focal spots were spatially overlapped, with the excitation focal spot nearly diffraction-limited and the STED spot enlarged ~1.5 times to ensure a more uniform illumination across the excitation focus. We realized confocal fluorescence detection, employing a fiber-coupled hybrid photomultiplier PMT (HPM-100-40, Becker&Hickl) and bandpass filter (HQ690/60, AHF). The STED beam intensity was modulated by a fast chopper CH (3501 Optical Chopper, New Focus) with rise and fall times of 30 μs at 4.5 Hz frequency, similarly to early experiments^22, 23^. The dynamics of fluorescence recovery after stopping the STED beam by the optical chopper allowed us to disentangle the irreversible bleaching and reversible fluorescence inhibition by de-excitation (Fig. 1c). The fast recovery component corresponds to the fraction of signal instantaneously silenced by stimulated emission. The slow component is related to translational diffusion of fresh molecules into the focal spot and its magnitude represents the STED-light-induced photobleaching in the focal region (see further discussion in the Supplementary Information).

### Photobleaching measurements

All measurements were carried out with a stationary focus (to avoid position drift of the scanning system, as often observed in long-term experiments with molecular layers) placed into dye solution samples to fulfill uniform chemical conditions. We chose to investigate four commercially available organic dyes in detail. These were: two standard red-emitting dyes for the STED laser wavelength applied in this work (ATTO647N, STAR635P), as well as two dyes with spectra shifted to the blue (ATTO590, STAR580). These dyes were obtained from ATTO-TEC (Siegen, Germany) and Abberior (Göttingen, Germany). The example spectral properties of both groups of dyes are presented in Fig. 1b. The fluorophores were dissolved in thiodiglycol (2,2′-thiodiethanol, TDE, S(CH_2_CH_2_OH)_2_, Sigma) as obtained from the bottle. The solvent was chosen such that its viscosity allowed for slow re-diffusion of fresh dye molecules into the focal spot region, and thus slow fluorescence recovery to compensate for bleached dyes. Long exposure to the STED light (~100 ms at a time) ensured to include photobleaching mediated by the triplet state (triplet lifetime *τ*_*tr*_ ≈ 1 μs−10 ms) or other long-lived dark states. All samples were enclosed between a concavity microscope slide and a standard cover glass sealed with nail polish. Each measurement was taken in steady-state conditions, with signals averaged over ~100 s. One such averaged data set consisted of three intensity traces, obtained separately (Fig. 1c): one for the excitation beam applied alone, one for excitation light together with the periodically blocked STED beam, and one for the modulated STED beam alone. All curves were normalized to the initial fluorescence signal registered for excitation light applied on its own (*C*_0_). From the characteristic levels *C*_*E*_, *C*_*ES*_, *C*_*S*_, the STED de-excitation (depletion of excited state) (*D*), bleaching (*B*) and STED-light-induced fluorescence (*SF*) can be derived as follows^11^:

1 $$D=1-\frac{({C}_{ES}-{C}_{S})}{{C}_{E}}$$

2 $$B=1-{C}_{E}$$

3 $$SF={C}_{S}$$

The excitation power was kept constant (30 μW) in the experiments described.

## Results

### STED de-excitation photon flux influence on “on”-”off” switching contrast and photobleaching. Temporal distribution of de-excitation photons: short de-excitation pulses (0.1–3 ps)

**Figure 2:**
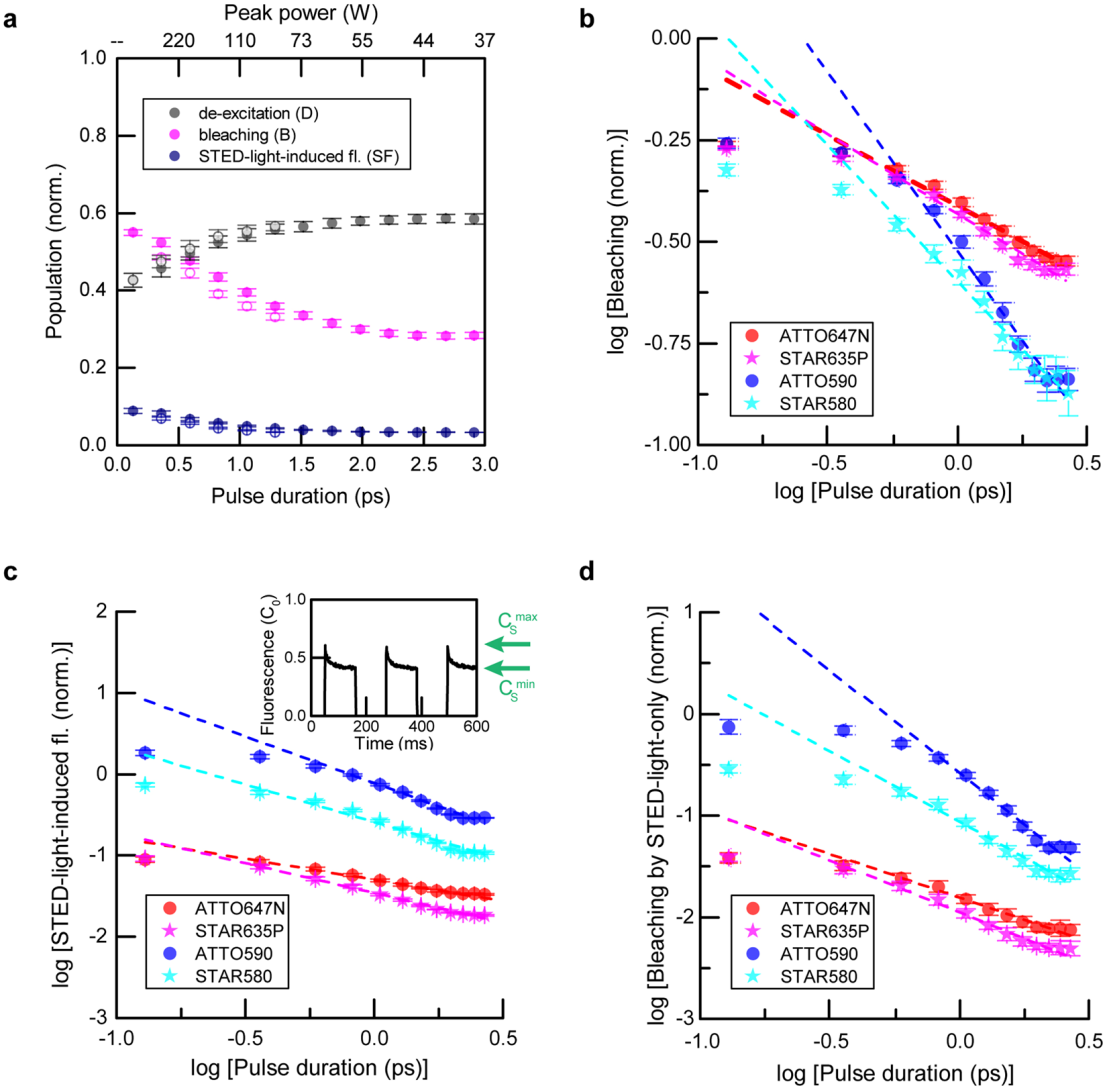
Pulse duration influence (0.1–3 ps) on de-excitation, bleaching and fluorescence induced by the STED beam at 750 nm. (**a**) Example ATTO647N: De-excitation, bleaching and STED-light-induced fluorescence vs. STED pulse duration. Hollow data points represent down-chirped STED pulses. (**b**–**d**) Bleaching (b), STED-light-induced fluorescence (c) and bleaching induced by the STED light on its own (d) vs. STED pulse duration for four dyes: ATTO647N, ATTO590, STAR635P and STAR580. Data is shown in log-log representation. The magnitude of bleaching by the STED pulse alone (d) was calculated from the reduction in the STED-light-induced fluorescence signal ((c), inset (example for ATTO590) – see text). Time-averaged STED power: 10 mW. All measurements were carried out for the same dye concentration (10 μM).

To characterize photobleaching, we investigated the influence of the pulse duration (τ) for short de-excitation pulses (0.13–3 ps), delayed slightly (7 ps) with respect to the ultrashort excitation pulse. All measurements were performed for the same dye concentration (10 μM) and de-excitation pulse energy (*E*_*p*_ = 0.125 nJ, STED power P = 10 mW). Results for ATTO647N are presented in Fig. 2a. For de-excitation pulses comparable in duration to vibrational relaxation of the excited state, the de-excitation efficiency decreases due to unproductive de-excitation/excitation cycles related to the high STED photon flux, before vibrational relaxation can occur^1^. With increasing pulse durations, de-excitation is more efficient. Our data indicates that bleaching is a nonlinear process, as it depends on the pulse duration as (*E*_*p*_ */τ*)^*b*^ *τ* ~ *τ* ^*−*(*b-1*)^, where *b* is the order of nonlinearity. The observed nonlinearity results from the interaction of excited molecules with photons that are applied to induce the de-excitation. Destructive chemical reactions often occur following optical transitions to higher (more reactive) excited states of the fluorophore. The fluorescence induced by the STED beam in this case (ATTO647N) is nearly negligible (typically ~5% of initial excitation). The observation of increased STED-light-induced fluorescence for short pulses (<1.5 ps) indicates both a reduction of excited-state depletion and higher involvement of two-photon absorption compared to the longer pulses.

The order of nonlinearity *b* for bleaching and the order of nonlinearity *sf* of STED-light-induced fluorescence with intensity can be retrieved as the line slopes in log-log representation (Fig. 2b,c). Analyzing the dependencies for different dyes gives respective values of the photobleaching order in the range *b* ≈ 1.4–1.9 and STED-light-induced fluorescence *sf* ≈ 1.5–2.2 (Table 1). For the red dyes ATTO647N and STAR635P, the SF signal was negligible (~5%). For this class of dyes, the major interaction between ground-state molecules and STED photons is by one-photon, Anti-Stokes linear absorption, which raises hot molecules to the first excited state. These results are in good agreement with the expected linear absorption cross-section at the used STED wavelength (~2∙10^−5^ of the maximal excitation absorption cross-section for ATTO647N) and the low average STED power (10 mW) used in the experiments. Since unwanted excitation by the STED beam at 10 mW is negligible (a few percent of initial excitation), most photobleaching processes are initiated by the interaction of already excited molecules with STED photons which did not perform the de-excitation within the excitation focal spot. For both red dyes, the bleaching shows a nonlinear dependence, with order of nonlinearity *b* ≈ 1.4. For the orange-emitting dyes ATTO590 and STAR580, the STED-light-induced fluorescence was comparable or higher than the excitation signal, and a meaningful de-excitation efficiency cannot be specified (Fig. 2c). For these two dyes (ATTO590, STAR580), the mechanism of STED-light-induced fluorescence was two-photon absorption (*sf* ≈ 2, Table 1), manifesting as a strong increase (~1/*τ*) of the STED-light-induced fluorescence signal with shortening pulse duration (Fig. 2c). The bleaching showed a higher order of nonlinearity than for the red-emitting dyes, as it is initiated by multiphoton excitation by the STED beam at 750 nm, which additionally populates the excited state. Moreover, for all four investigated dyes, the SF signal indicated strong bleaching due to the STED light alone acting on the molecules (defined as the difference in signal between maximal and minimal induced fluorescence (*C*_*S*_^max^–*C*_*S*_^min^) during illumination by the STED light (Fig. 2c inset and Fig. 2d). Photobleaching due to the STED beam acting alone exhibited a higher-order dependence than the bleaching of already excited molecules (by the excitation light), for all four dyes examined (Fig. 2d and Table 1). In this case, the effective order of photobleaching depends both on the order of the STED-light-mediated excitation mechanism (e.g. linear vs. two-photon) and on the order of the effective photobleaching rate starting from the first excited state with respect to intensity (related to the dominant photobleaching pathway, e.g. via the first triplet, or sequential absorption steps to higher states, see below). Interestingly, we observed a noticeable dependence of bleaching and STED-light-induced fluorescence on the chirp of the de-excitation light (Fig. 2a hollow points, see Supplementary Information). Chirped pulses are characterized by temporal delays of different spectral components along the ultrashort pulse. For all tested dyes and peak intensities, down-chirped STED pulses (led by the bluer components) showed ~25% lower SF and ~10% lower bleaching in comparison to up-chirped de-excitation pulses in this short-pulse regime. Within the measurement error, fluorescence de-excitation was similar in both cases. The error of measurement in the tabulated data represents the standard error of the line fit in the pulse duration range >500 fs.

**Table 1:** Orders of nonlinearity for STED-light-induced fluorescence, photobleaching and photobleaching by STED light on its own, as measured by varying the STED pulse duration over the range 0.1–3 ps.

	STED-light-induced fluorescence (*sf*)	Bleaching (*b*)	Bleaching by STED only
ATTO647N	1.52 ± 0.02	1.39 ± 0.02	1.86 ± 0.05
ATTO590	2.15 ± 0.07	1.89 ± 0.06	3.02 ± 0.08
STAR635P	1.73 ± 0.02	1.35 ± 0.02	2.03 ± 0.04
STAR580	1.92 ± 0.05	1.66 ± 0.04	2.40 ± 0.07

**Figure 3:**
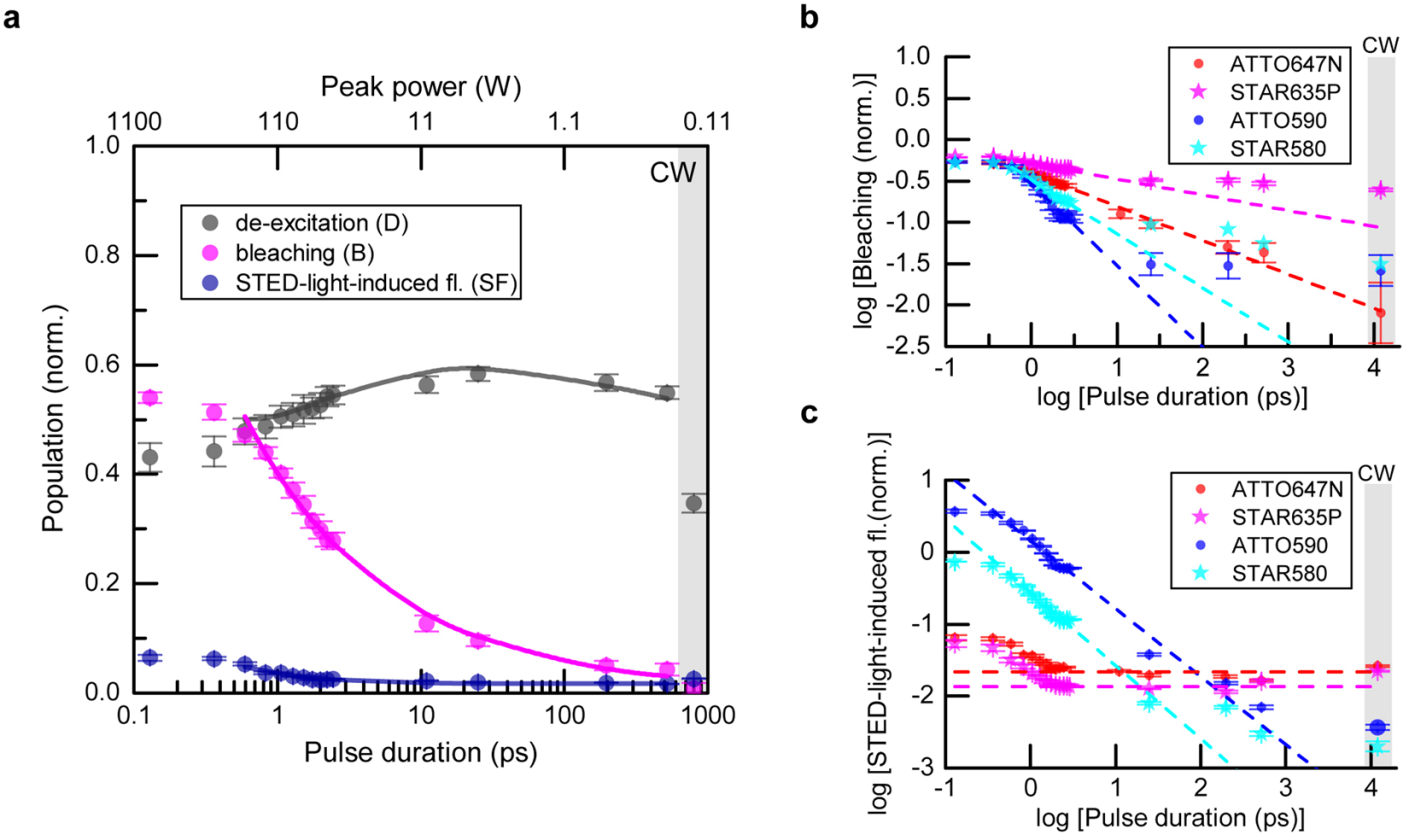
Pulse duration influence on de-excitation, bleaching and fluorescence induced by the STED beam at 750 nm. (**a**) De-excitation, bleaching and STED-light-induced fluorescence vs. pulse duration for ATTO647N. CW represents measurements for continuous-wave STED laser operation. The solid lines represent values calculated within the photobleaching model (Supplementary Information). (**b**) Bleaching vs. STED pulse duration for different dyes. The dashed lines represent the orders of photobleaching, with values already determined for pulses of 0.1–3 ps (Fig. 2). Deviations from this scaling indicate the dominant involvement of a different photobleaching pathway in these pulse-duration regimes (see text). (**c**) STED-light-induced fluorescence vs. STED pulse duration. For organic dyes typically used with the laser wavelengths applied in this study (ATTO647N, STAR635P), STED-light-induced fluorescence is mainly constant (flat dashed lines). For the orange-emitting dyes studied (ATTO590, STAR580), significant fluorescence emission induced by the STED beam occurs via two-photon absorption. Time-averaged STED power: 10 mW (E_p_ = 0.125 nJ). Measurements were performed for a low dye concentration (~0.5–10 μM).

Tabulated coefficients are retrieved from line slopes presented in Fig. 2b–d, also shown in Fig. 3b,c. Time-averaged STED power: 10 mW (E_p_ = 0.125 nJ).

### Longer de-excitation pulses (>10 ps)

We extended our investigations to longer durations of de-excitation pulses (Fig. 3). Here, the delays between excitation and de-excitation pulses were adjusted to approximately the STED pulse duration, ~*τ* (FWHM), to maintain the highest possible de-excitation (see Supplementary Information). The STED pulse energy was fixed during all measurements as before (*E*_*p*_ = 0.125 nJ), concentrations of the dye solutions were in the range ~0.5–10 μM. While de-excitation and STED-light-induced fluorescence were nearly constant for ATTO647N over a large range of pulse durations (10–500 ps) (Fig. 3a), the non-recovered fraction of signal after STED-beam exposure representing bleaching, *B*, showed a strong dependence on pulse duration (Fig. 3a,b), being reduced from *B* ≈ 0.5 for ~1 ps pulses to *B* ≈ 0.01 or below for CW de-excitation. The least bleaching was observed for CW, which also produced the lowest de-excitation: under CW conditions only a fraction (~30%) of the STED-light photons act on the excited-state molecules and can potentially switch off fluorescence at the 80 MHz repetition rate of excitation pulses, acting within the decay of spontaneous emission (~3.5 ns). As for the example ATTO647N (Fig. 3a), the de-excitation efficiency was nearly constant over a broad range of pulse durations also for the other investigated dyes (not shown). For the orange-emitting dyes (ATTO590, STAR580), the STED-light-induced fluorescence showed a clear signature of two-photon absorption from STED pulses over the entire range of pulse durations, while both red-emitting dyes (ATTO647N, STAR635P) showed STED-light-induced fluorescence at a largely constant level for long pulses (25 ps to CW) (Fig. 3c), indicating linear absorption as the dominant STED-light-induced excitation mechanism.

Despite these differences in interaction between ground-state molecules with STED-light photons, photobleaching clearly depends on the peak intensity for all dyes, and the peak intensity should be as low as possible to reduce high-order photobleaching (Fig. 3b), while also seeking to maintain sufficient de-excitation. Notably, for ATTO647N, the bleaching followed a constant scaling law of order ~1.4 over the whole range of pulse durations (Fig. 3b). However, for the three other dyes examined, the bleaching showed different dependencies than expected based on the previous measurements in the short-pulse regime (Fig. 3b, dashed lines and deviations therefrom). This suggests that different photobleaching mechanisms^28, 29^ (low vs. high order) play a limiting role at different (regimes of) peak powers, with different contributions of multi-step absorption processes of STED light vs. the constant bleaching (e.g. intensity-independent bleaching mediated by the build-up of the first triplet state or other dark states).

While there were differences in the absolute magnitudes of photobleaching in comparison to the data presented above, the order of photobleaching *b* was found to be similar in both measurement series (compare Figs 2b and 3b). The offset between them can be explained by different low-order photobleaching (e.g., photobleaching rate independent of intensity, related, for example, to the triplet state T_1_). The difference likely arose due to a change of the solvent (TDE had to be taken from a different bottle for the experiments of Fig. 2 vs. those of Fig. 3, with possibly slightly different oxygen content). We observed no difference in the photobleaching order with dye concentration changes (1−10 μM) between the measurement series.

### Power dependencies

**Figure 4:**
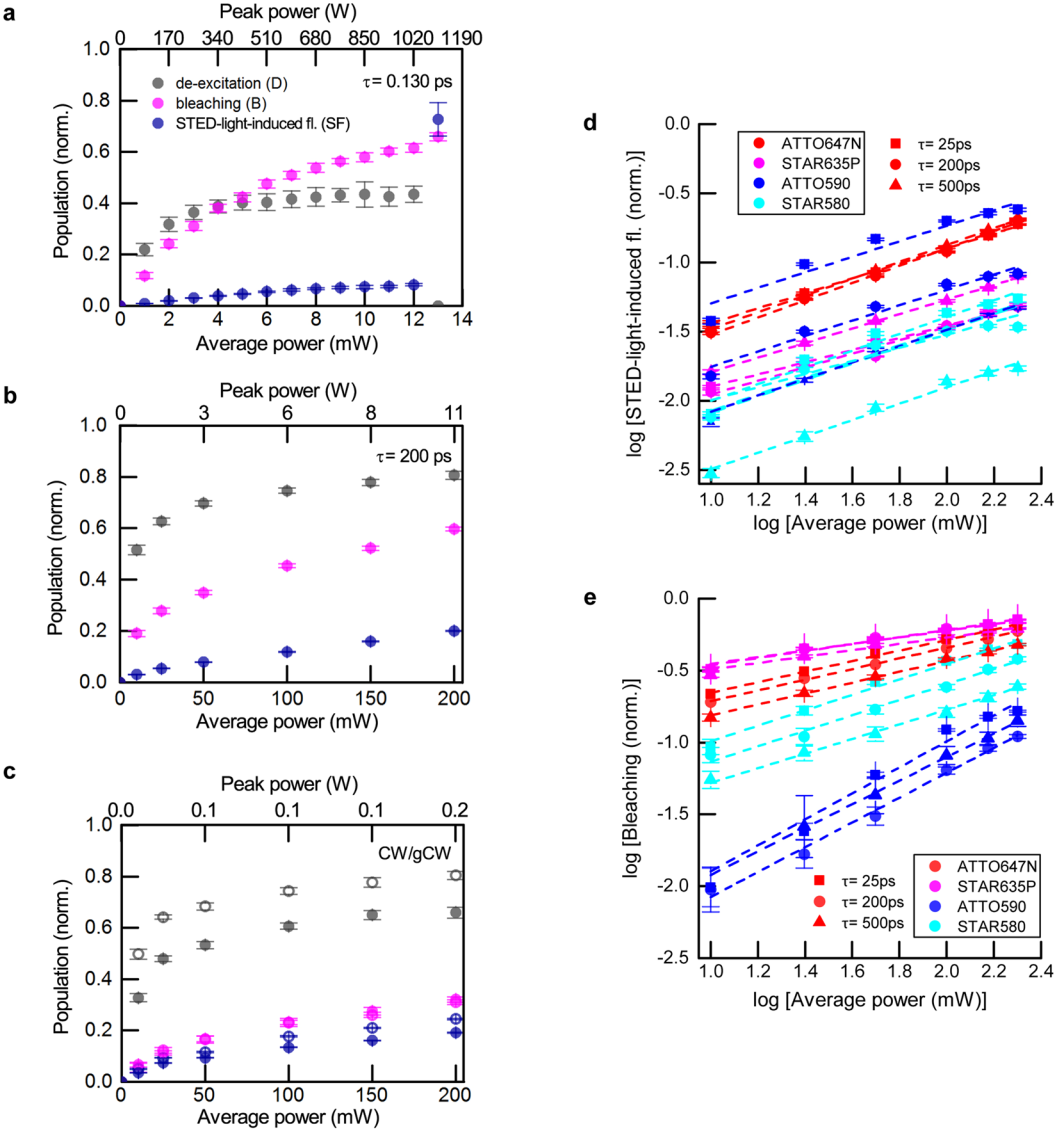
Influence of STED power on de-excitation, bleaching and fluorescence induced by the STED beam. (**a**–**c**) De-excitation, bleaching and STED-light-induced fluorescence vs. time-averaged power for Gaussian STED pulses of 130 fs (**a**), 200 ps (**b**) duration, and for CW STED (**c**). The hollow circles in (c) indicate CW STED operation with time-gating of detected fluorescence (see text). Measurements in (a–c): ATTO647N. (**d**) STED-light-induced fluorescence vs. power for different dyes and different pulse durations. (**e**) Bleaching vs. power for different dyes and different pulse durations. Note the log-log representation of data in (d,e).

The measurements described above were performed for much lower average power (10 mW) than typically applied in standard STED operation for high resolution levels (>100 mW) at this repetition rate. The reason is that we desired to perform the comparative study for the whole range of pulse durations. Yet, at just slightly higher average powers (~13 mW), we had observed a drastic increase in STED-light-induced fluorescence (likely as a result of photolysis) for the shortest, ~130 fs, STED pulses with peak power ~1.2 kW (Fig. 4a, ATTO647N), a pulse length regime we desired to include in the series. To explore bleaching under more realistic STED conditions we further examined the influence of the STED photon flux by varying the average power for a set of different pulse durations (Fig. 4). Increasing the pulse duration from 130 fs to 200 ps or CW allowed to apply more de-excitation photons without increasing bleaching (Fig. 4b,c). Regardless of the nonlinear nature of photobleaching shown above for measurements with varied pulse duration (b > 1, Figs 2 and 3), measurements as a function of STED power *P*_*STED*_ revealed a sublinear bleaching dependency ~*P* ^*b*^ with *b* < 1 (Fig. 4a–c,e; Table 2), even for de-excitation pulses as short as 130 fs. As previously, we determined the orders of nonlinearity *b* and *sf* for long pulse durations (~25 ps, ~200 ps, ~500 ps) by line fits in log-log representation for all investigated dyes (Fig. 4d,e). Surprisingly, *b* and *sf* did not vary significantly over the investigated range of pulse durations. Averaged values for all dyes are presented in Table 2, with errors of measurement estimated from maximal and minimal deviations from the line fit (Fig. 4d,e). The sublinear power dependence of photobleaching is related to the drastic decrease in occupation of the excited state with increasing STED power (pulse energy), which masks the underlying mechanism(s) of photobleaching as a function of photon flux (instantaneous intensity).

**Table 2:** Orders of nonlinearity of bleaching and STED-light-induced fluorescence, as measured by varying the STED average power.

	STED-light-induced fluorescence (sf)	Bleaching (b)
ATTO647N	0.59 ± 0.04	0.37 ± 0.01
ATTO590	0.54 ± 0.06	0.9 ± 0.1
STAR635P	0.49 ± 0.04	0.23 ± 0.01
STAR580	0.59 ± 0.04	0.53 ± 0.03

Tabulated coefficients are retrieved from line slopes of the lines presented in Fig. 4d,e.

We observed similar photobleaching behaviours for ~200 ps Gaussian pulses and for CW, however, to maintain the same de-excitation efficiency, the STED average power has to be ~five-fold higher in the second case. This mandatory power increase results in increased STED-light-induced fluorescence for the CW case, due to increased linea absorption of STED photons. Similar de-excitation to 200 ps STED pulses, at the same power level, can be obtained by applying gated detection (gCW)^25, 26^, at the expense of detected fluorescence signal (Fig. 4c, hollow points). For the gCW measurements, we registered the signal starting at ~*τ*_*fl*_ /2 after excitation, where *τ*_*fl*_ = 3.5 ns denotes the fluorescence lifetime. This meant that the fluorescence signal was reduced by ~40% by gating. Note that, for gCW, the STED-light-induced fluorescence signal, as detected with gating, is higher than for CW STED at the same power. The determined bleaching coincided for both cases. Maintaining the same signal for dim samples requires longer dwell times and would increase photobleaching due to increased exposure times.

### Influence of STED photon flux in imaging

**Figure 5:**
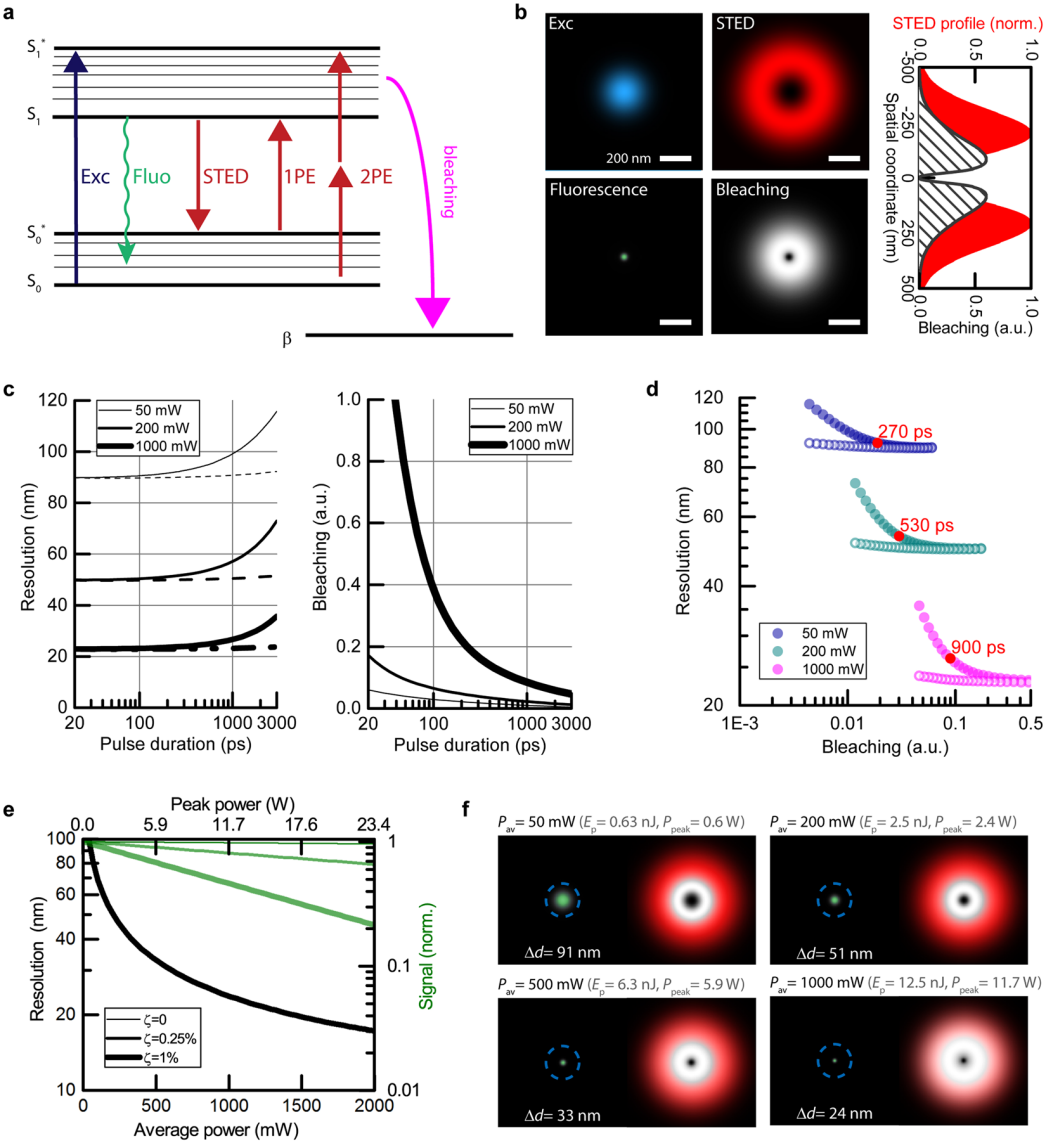
Simulation of the STED performance at 750 nm in imaging (based on parameters derived from measurements with ATTO647N). (**a**) Simplified model of de-excitation, bleaching and STED-light-mediated 1-photon (1PE) and 2-photon (2PE) excitation processes. (**b**) Focal-plane excitation (Exc) and STED spatial profiles (top). Resulting fluorescence and probability of photobleaching (bottom), shown here for a STED pulse duration of τ = 200 ps and average power P = 450 mW (80 MHz repetition rate). (**c**) Resolution and bleaching as a function of pulse duration for three time-averaged STED powers. The dashed lines represent gated detection. (**d**) The same data as in c presented as resolution vs. bleaching. The lowest bleaching and the highest resolution can be obtained by adjusting the STED pulse duration and applying gated detection (hollow points, t_gate_ = τ). (**e**) Influence of residual STED intensity ζ at the targeted coordinate (intensity minimum) on resolution and fluorescence signal amplitude for three different levels: 0, 0.0025 and 0.01 of doughnut crest intensity. STED pulse duration: τ = 1000 ps, gated detection t_gate_ = τ. 1000 mW at 80 MHz repetition rate (this simulation) correspond in terms of pulse energy and peak power to 250 mW at 20 MHz, a common repetition rate for STED with nanosecond pulses. Note that the three black curves (resolution) overlap. (**f**) Spatial distribution of fluorescence (green), normalized probabilities of photobleaching (grey scale, with highest photobleaching in white) and normalized STED beam profile as reference (red) for different average STED powers (STED pulse duration: τ = 1000 ps). The stated resolution values Δd represent the respective FWHM of the fluorescence profile.

We modelled the influence of the STED photons on the resulting super-resolution image by simplifying the involved molecular electronic states to a standard 4-level system (Fig. 5a and Supplementary Information). We chose this system as it described the de-excitation efficiency over the whole investigated temporal range, including population-inversion for short pulses (see reduction in the de-excitation *D* for *τ* < 5 ps in Fig. 3a). To include photobleaching, we inserted an additional state (β) corresponding to irreversibly bleached molecules. We assumed that photodestruction occurs by interaction of excited molecules (S_1_*, S_1_ states) with the STED photons, i.e., molecules in the ground state (S_0_*, S_0_) are chemically stable, and the bleaching driven by the excitation light is negligible. Initially, all molecules are in the ground state and can be transferred to the S_1_* state by linear absorption of photons (from the excitation or STED pulse) or by two-photon absorption of STED photons. Then, after vibrational relaxation to S_1_, molecules can relax to S_0_* by emitting fluorescence, or be transferred to the state S_0_* by stimulated emission, or photobleach to end up in β. The paths taken by the excited-state fluorophore depend on the instantaneous STED intensity and probabilities of each process, which have to be found experimentally. We chose to model the behaviour of ATTO647N, as the data for this dye suggested a single scaling of photobleaching over the whole range of pulse lengths (peak powers), as ~*I*_*STED*_^1.4^ (Fig. 3b). To find the optimal parameters which simultaneously describe the three experimental curves (*D*, *B*, *SF* in Fig. 3b) in a global fit analysis, we varied the STED pulse durations to match measurements and numerically calculated the expected values for de-excitation, bleaching and STED-light-induced fluorescence in our confocal detection scheme, for 3D spatial distributions of excitation and STED foci (Supplementary Information). We inferred the following parameters: stimulated emission cross-section *σ*_*STED*_ = 4.8∙10^−18^ cm^2^, cross-section for linear excitation by the STED beam *σ*_*1PE*_ = 3.5∙10^−21^ cm^2^, cross-section for two-photon excitation by the STED beam *σ*_*2PE*_ = 3.5∙10^−50^ cm^4^ = 3.5 GM, and an effective photobleaching coefficient from the excited state *k*_1_ = 5.2∙10^−10^ Hz. The intensity-dependent photobleaching rate *k* was defined as *k* = *k*_1_ · (*I*/*I*_0_)^*b*^, with *b* = 1.4 and *I*_0_ = 1 W/m^2^. The quality of the fit with respect to experimental data is seen in Fig. 3a (solid lines).

By introducing the spatial intensity distribution for the Gaussian excitation and doughnut-shaped STED profile in the focal plane, the effective fluorescence point spread function (PSF) and the spatially dependent probability of photobleaching were numerically calculated in the focal-plane, based on the inferred parameters (Fig. 5b,f). We defined the resolution as the full width at half maximum (FWHM) of the fluorescence profile, and computed photobleaching as proportional to the integral over its spatial distribution (Supplementary Information). To enable a fair comparison between different pulse durations, we chose the most favourable time delay between excitation and STED pulses, which was calculated numerically to be within FWHM/2 and FWHM of the Gaussian STED pulse duration for pulses longer than 100 ps (Supplementary Information).

We modelled the resolution and photobleaching as a function of Gaussian pulse duration for different time-averaged STED powers, with results for 50 mW, 200 mW and 1 W shown in Fig. 5c. Differences in de-excitation pulse duration affect both the de-excitation efficiency and the bleaching, and we thus additionally present resolution as a function of photobleaching (rather than separate duration dependencies) to facilitate comparisons (Fig. 5d). The resolution, similarly to de-excitation (its enabling process), is independent of pulse duration over a broad range of pulse durations (20–200 ps), as stimulated emission is a linear process in pulse energy, i.e. linear with the number of photons applied. In contrast, the bleaching shows a strong dependence on pulse duration. For example, by changing the duration from 200 ps to 1000 ps at 200 mW average STED power, resolution drops by only ~10%, with a reduction of bleaching by more than 50%. Optimal pulse duration (the best compromise between resolution and bleaching) depends on the available average power (approximately, the red points in Fig. 5d). Under low average power conditions (with low intensity-dependent bleaching, resolution ~70 nm), short pulses are preferable (~270 ps), as they provide the most efficient de-excitation from the excited state in non-gated STED operation. Higher-power applications are limited by photobleaching, and long de-excitation pulses (~ns) provide a better trade-off. Applying long pulses combined with gated detection (Fig. 5c, dashed lines; Fig. 5d, hollow circles) allows always to maintain the highest resolution and low bleaching, at the expense of a reduction of registered fluorescence signal. It is important to note that high STED powers require particularly low residual intensity at the coordinate targeted by the intensity minimum (Fig. 5e). For example, for a relative residual intensity of *ζ* ≈ 1% at the intensity minimum (compared to the doughnut crest) and resolution level of ~20 nm, nearly 70% of the initial fluorescence amplitude is lost due to de-excitation of fluorophores directly at the doughnut minimum^30^.

**Figure 6:**
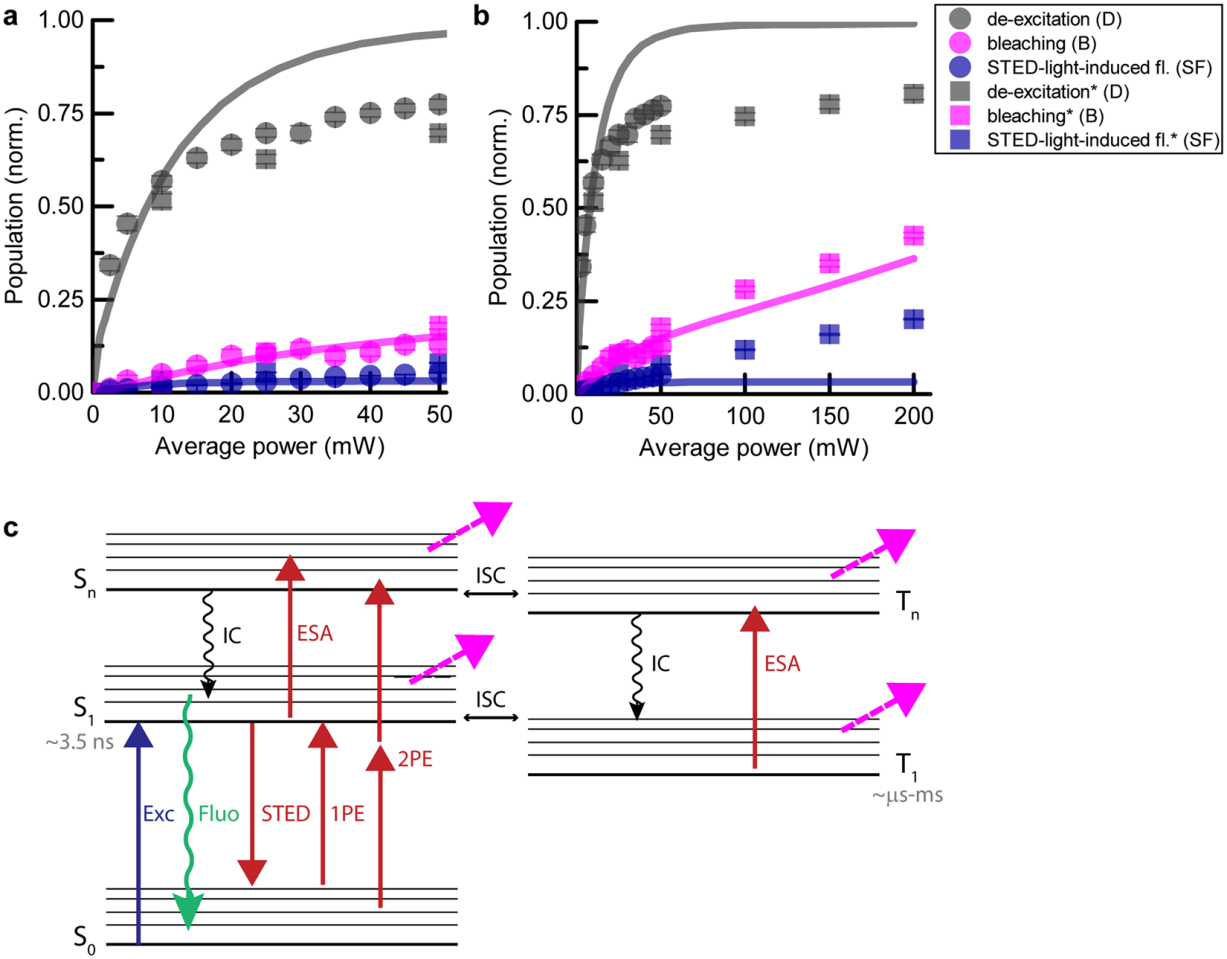
Limitations of the photobleaching model in explaining the data at high STED powers (ATTO647N) (see text). (**a**,**b**) Comparison between experimental and modelled data for de-excitation, bleaching and STED-light-induced fluorescence vs. STED power. (a) represents a subset of the data shown in (b). Circles and squares correspond to two independent measurement series). (**c**) Extended model of states possibly involved in photobleaching^32^, including an additional excited singlet state S_n_, and two triplet states T_1_ and T_n_. ISC: intersystem crossing; IC: internal conversion; ESA: excited-state absorption processes.

Our model quite successfully described de-excitation, photobleaching and STED-light-induced fluorescence as a function of STED pulse duration (Fig. 3a). Nonetheless, applying the same model to measurements as a function of the average STED power led to inconsistencies (Fig. 6a): the measured de-excitation *D* saturated at lower values and the measured STED-light-induced fluorescence *SF* was significantly higher than expected from the model based on the previously determined parameters for ATTO647N. Nonetheless, for all measurements, photobleaching shows the same ‘slope’ as a function of STED power and is described by the proposed model (showing the same scaling with STED power). As mentioned above, for different ATTO647N samples with solvent from either bottle, the registered photobleaching shows different constant offset (low-order photobleaching, compare Figs 4b and 6a squares), which was corrected in Fig. 6 by subtracting a constant value (~0.2) from one experimental data set (Fig. 6a squares).

The observed differences for de-excitation and STED-light-induced fluorescence may stem from the fact that other, additional electronic states are involved (Fig. 6b), which become relevant at higher STED powers. It is important to consider at least three other states: the higher excited state S_n_, and the lower and higher triplet states T_1_,T_n_^31, 32^. The effects of bleaching from singlet states S_n_ could be subsumed to processes from S_1_ (due to very fast relaxation times (~ few ps) by internal conversion IC). However, it is challenging to include the involvement of triplet states in numerical simulations, due to their much longer time scale and ambiguities that arise if several new states, and therefore new parameters, are postulated. The observed increased STED-light-induced fluorescence at high average powers might be explained by delayed fluorescence: molecules trapped in the lower triplet state T_1_ can be directly excited by STED light to higher triplet states T_n_ and then, with some probability (defined by intersystem crossing ICS) come back to the electronic singlet system. The time scale of this process is related to the lifetimes of higher triplet states; for instance, the typical lifetime of the second triplet state T_2_ is ~100 ns. A five-level system, incorporating states as shown in Fig. 6c, is sufficient to describe photobleaching data in microscopy^31, 32^. Nevertheless, the number of free parameters makes it less intuitive. Moreover, the influence of the photobleaching from the triplet or other dark states should be significantly reduced by application of low-repetition rate lasers and triplet quenchers, in which case the contribution of high-order photobleaching would become dominant. Note that the influence of the triplet system could in principle be elucidated by time-reversal of the excitation and STED pulse, respectively, and this was studied in prior work^11^. By applying repetition rates below MHz, the involvement of all triplets can be reduced^12, 13^ at the expense of significantly increased measurement time to collect fluorescence signal. Reduction of the repetition rate from 80 MHz to e.g. 10 MHz should reduce the build-up of the second triplet state (with lifetime ~100 ns) and reduce photobleaching associated with this state. The same outcome can be obtained by very fast scanning to allow dark-state relaxation^5, 14^.

It is important to note that higher resolution demands higher pulse energies (sufficiently large numbers of de-excitation photons), and careful attention is required in choosing the fluorophore. For standard dyes such as ATTO647N, the linear absorption cross-section of the STED light is five orders of magnitude lower than the absorption cross-section at the excitation wavelength (Supplementary Information). This means that applying an excitation power of ~3 μW and a STED power of ~300 mW will result in comparable numbers of molecules excited per unit volume by both beams. The difference is that the STED light also serves to de-excite molecules – meaning that those excitation events are followed by *de*-excitation and thus do not contribute to the measured signal. Additionally, confocal detection rejects signal from outside the excitation-light spot. The underlying interaction (STED light with molecule), however, causes major photobleaching in the peripheral region surrounding the targeted coordinate, i.e. predominantly at the doughnut crest (Fig. 5b, Supplementary Information) for high STED average powers. Shifting the STED wavelength to even longer wavelengths and/or using dyes with spectra shifted to shorter wavelengths should thus be beneficial for STED implementations with very high average powers where linear absorption of STED-light photons initiates further excitation steps and thus photobleaching. To illustrate the behaviour of a different (orange) dye, modelling for ATTO590 is discussed in the Supplementary Information (see Supplementary Fig. S8 and Supplementary Table S4). Careful studies are still needed to establish the spectroscopic properties of other states, and in particular to shed more light on the process of excited state absorption^33^ (ESA) and its involvement at the STED wavelength for the dyes in use.

## Discussion

In STED nanoscopy, efficiently de-exciting fluorophores outside the targeted coordinate is the key to super-resolution. High resolution requires to deliver the necessary numbers of photons while fluorophores reside in the excited state. Sufficient intensity close to the targeted coordinate is accompanied by much higher excess exposure at the doughnut crest. It is especially these high de-excitation light intensities which lead to unwanted photon absorption events and photobleaching. Depending on the photon flux acting on the excited fluorophores, two different photobleaching regimes can generally be distinguished for constant pulse energy: bleaching that is independent of the intensity (low-order photobleaching), and intensity-dependent bleaching which is associated with both singlet and triplet higher excited states (high-order photobleaching). One low-order photobleaching pathway is by reactions undergone by fluorophores trapped in the first triplet state, or other dark states. High-order bleaching occurs because of the more reactive nature of the higher excited states, which are populated by multiphoton or multiple sequential absorption steps.

In this study, we provide quantification of a number of experimental aspects in STED microscopy. Our data corroborate previous strategies that had been followed based on physical intuition, particularly the realization that longer pulses have advantages in minimizing photobleaching. The results highlight subtle but important differences even for dyes that have very similar emission spectra (STAR635P vs. ATTO647N), suggesting that different optical strategies, tailored to the particular dye and its environment during imaging, will best mitigate bleaching.

At low STED powers (e.g. ~10 mW for ATTO647N), the de-excitation photons predominantly interact with fluorophores to induce stimulated emission, which serves to transfer them to the non-reactive ground state and thus reduce photobleaching in comparison to the excitation light acting alone^14, 34^. As the STED intensity is increased for higher resolution, other interactions with the STED light cannot be neglected any more: absorption of STED-light photons, which raise the fluorophores to either the first excited singlet and/or higher excited singlet and triplet states, becomes more probable. Occupancy of each of these excited states contributes to the overall magnitude of bleaching. We argue that the exact chemical mechanisms need not strictly be known in detail to identify optimal parameters for STED imaging (pulse energy, pulse duration, repetition rate). Rather, the overall dependence of photobleaching on STED photon flux needs to be characterized, and this was the approach taken in this study. In this work, we verified the reduction of high-order photobleaching by lowering the peak intensity (using longer pulses), whereas low-order photobleaching is not strongly affected by pulse duration, and other strategies such as the aforementioned dark-state relaxation schemes^13, 14^ and triplet quenchers hold more promise to reduce this contribution.

In our study, photobleaching measurements as a function of STED intensity, by varying the time-averaged power (Fig. 4), resulted in sub-linear dependence (orders < 1), but measurements in which the pulse duration was varied (Figs 2 and 3) showed a super-linear dependence (orders > 1). This can be explained as in measurements vs. power the numbers of delivered photons are varied, affecting both the de-excitation and bleaching in ways that are difficult to disambiguate. In measurements vs. pulse duration, the energy per pulse was constant (i.e., a fixed number of de-excitation photons), with little effect on the de-excitation (Fig. 3a), meaning that these measurements isolate the photobleaching behaviour, as they are not compromised by altered occupancies of the excited state.

A significant reduction of photobleaching was observed for increasing de-excitation pulse durations, which implies a high-order photobleaching mechanism (orders in the range 1.4 to 1.9) for all four investigated dyes at high peak powers of 10 W and above. Our data therefore reveal the involvement of high-order photobleaching at the currently applied STED peak powers^2, 8^. For very short pulses (0.13–3 ps), photobleaching by the STED beam action alone was noticeable. Moreover, for de-excitation pulses shorter than ~2 ps, it was impossible to obtain efficient de-excitation due to numerous unproductive de-excitation/excitation cycles caused by the very high photon flux, as expected theoretically^1^ and seen in early experiments^22^. The number of these cycles was reduced to some extent by applying pulses with a down-chirp. The down-chirp not only reduced the STED-light-induced fluorescence but also the photobleaching (compared to the standard up-chirped pulses): effectively, fluorophores will be found in the reactive excited state for a shorter time (Supplementary Information). The influence of chirp for long de-excitation pulses is likely minor, as it depends on the vibrational lifetime (~1 ps) and may only become relevant at high photon fluxes.

For lower photon fluxes (peak powers < 10 W), three of the four dyes exhibited almost intensity-independent photobleaching for constant pulse energy. We found that ATTO647N followed a single scaling of bleaching for all peak powers (order ~ 1.4), which may be explained by low ISC and thus negligible bleaching from the triplet system, in line with previous observations. The bleaching from the whole singlet system effectively scales like bleaching from the first singlet state, as the higher singlet states’ lifetimes are shorter by orders of magnitude, and their bleaching contributions can be subsumed into bleaching from the first excited singlet state (with one effective rate, dependent on intensity due to involvement of the higher states).

Our results highlight the importance of the STED-light absorption. It leads to a small contribution to fluorescence emission induced by the STED beam. The collected signal is typically negligible due to efficient de-excitation and confocality of detection, but even the finite occupation of the excited state under a strong photon flux, with many molecules repeatedly transferred between the ground and excited electronic state, results in significant photobleaching. Moreover, the registered dependence of photobleaching vs. STED intensity, and thus optimal pulse duration, is strongly affected by the mechanism of STED-light excitation of fluorophores (one- vs. two-photon process). This mechanism of excitation is difficult to access experimentally due to the involvement of stimulated emission. Based on modelling (Fig. 3a), we conclude that two-photon absorption of the STED light by ATTO647N is negligible at the peak powers accessed in all STED experiments to date (up to ~20 W, Supplementary Fig. S9), and that the dominant mechanism of its STED-light-induced fluorescence is by linear absorption. For STED implementations with de-excitation pulses of a few hundred picoseconds, resolutions down to ~30–40 nm have been reported for red dyes and nano-beads (Supplementary Fig. S9). A possible explanation why further resolution increases, requiring higher STED photon flux, have not been realized with sub-nanosecond pulses, lies in the pronounced intensity-dependent photobleaching contribution under STED conditions (as experiments were not power-limited). In contrast, current STED implementations with ~1 ns pulses have allowed to reach the ~20 nm resolution level by delivering more photons at the same peak power.

The majority of bleaching for ~1 ns pulses with red dyes at high STED pulse energies is expected to be mediated by one-photon absorption events of the STED light (based on the room-temperature absorption cross-section at the STED wavelength 750 nm). This means that even the outermost parts of the STED doughnut drive bleaching (Fig. 5f and Supplementary Fig. S7c). The definition of the intensity zero (minimum) becomes even more important in order not to diminish the signal by off-switching at the targeted coordinate (Fig. 5e). Longer dwell times to accumulate more signal are accompanied by increased bleaching.

We have shown the spatial dependence of bleaching with respect to the targeted coordinate (Fig. 5b,f), which may prompt other insights. For example, applying a scanning scheme like MINFIELD^17^ will favour short durations of de-excitation pulses, as high-order photobleaching is already reduced by avoiding the exposure of molecules to the high intensity at the doughnut crest and periphery.

In summary, our experiments confirm that optimal de-excitation occurs for STED pulse durations of few tens of picoseconds, as molecules are switched off quickly before spontaneous relaxation by fluorescence takes place. Such comparatively short pulses, while featuring the best de-excitation, come with increased high-order bleaching, which plays a significant role in STED at high intensities, as our measurements indicate. High-resolution STED requires sufficient photon flux in the direct vicinity of the targeted coordinate to define the “on”-“off” contrast. This causes high-order photobleaching associated with excited states of the fluorophore, mainly outside the target coordinate. High-order bleaching can be controlled by providing the de-excitation photons in longer STED pulses, a strategy pursued over the past years of STED development. The success of this approach depends on the magnitude of low-order photobleaching, for which triplet-quencher or dark-state relaxation strategies exist. The application of longer STED pulses will have to be combined with suitable gating to maintain maximal resolution. Based on our experimental data, the recently shown superior resolution for long, nanosecond STED pulses can be rationalised, as it reduced the high-order bleaching.

## Electronic supplementary material

Supplements
