# Supplementary material for: Photobleaching in STED nanoscopy and its dependence on the photon flux applied for reversible silencing of the fluorophore
Source: Sci Rep. 2017 Sep 12;7:11354. doi: 10.1038/s41598-017-09902-x (PMC5595794; doi:10.1038/s41598-017-09902-x)
Supplement: Supplementary file 1 — Supplements [file 41598_2017_9902_MOESM1_ESM.pdf]

Supplementary Information

Photobleaching in STED nanoscopy and its dependence on the photon flux applied for reversible silencing of the fluorophore

Joanna Oracz[1,2,*], Volker Westphal[1], Czesław Radzewicz[2], Steffen J. Sahl[1], and Stefan W. Hell[1,3,*]

[1]Max Planck Institute for Biophysical Chemistry, Department of NanoBiophotonics, Am Fassberg 11, 37077 Göttingen, Germany
[2]University of Warsaw, Faculty of Physics, Pastera 5, 02-093 Warsaw, Poland
[3]Max Planck Institute for Medical Research, Department of Optical Nanoscopy, Jahnstr. 29, 69120 Heidelberg, Germany
[*]Correspondence should be addressed to S.W.H. (shell@mpibpc.mpg.de) or J.O. (joracz@mpibpc.mpg.de).

Contents

1 Temporal shaping of STED pulses **S2**
1.1 Short pulse duration regime ($0.13-3$ ps) . . . S2
1.2 Long pulse duration regime ($10-500$ ps) . . . S5
2 Data analysis **S5**
3 Influence of the STED pulse chirp **S6**
4 Simulation of de-excitation, bleaching and STED-light-induced fluorescence for ATTO647N **S8**
4.1 3D fitting procedure to experimental data . . . S8
4.2 2D STED microscope simulation . . . S12
4.3 Resolution scaling with STED power . . . S12
4.4 Optimal delay between excitation and STED pulse . . . S12
4.5 Bleaching at different STED powers . . . S13
5 Simulation of de-excitation, bleaching and STED-light-induced fluorescence for ATTO590 **S14**
6 Comparison: Resolution in STED microscopy reported for red dyes and fluorescent beads **S16**
Supplementary References **S17**

1 Temporal shaping of STED pulses

1.1 Short pulse duration regime (0.13−3 ps)

Characterization of the STED pulses at the back aperture of the objective lens

To obtain roughly transform-limited ultrashort pulses at the back aperture of the objective lens, we aligned the home-built pulse compressor to measure the shortest possible pulse duration (∼130 fs, data not shown) with a commercial autocorrelator (pulseCheck, APE). Next, we characterized the spectral phase introduced by the home-built pulse shaper. We compared the phase shift imparted at the spatial light modulator (SLM) on a femtosecond pulse with an unmodified replica of the same pulse ("reference pulse") using Fourier transform spectral interferometry (SI)[1]. The relative delay between two pulses t was controlled by the optical delay line (DL) placed in one of the arms of a Mach-Zehnder interferometer (Supplementary Fig. S1a). We registered the interference spectrum (S_{SI}) of the pulses on a high-resolution spectrometer (500M, Spex), to resolve the high-frequency components over the narrow laser bandwidth (Supplementary Fig. S1b, FWHM≈8.5 nm). The registered spectrum S_{SI} is described by:

$$S_{SI}(\omega) = 2S(\omega)\big(1+\cos(\Delta\varphi+\omega t)\big) \tag{1}$$

where $S(\omega)$ is the spectral amplitude of the reference and shaped pulse, $\Delta\varphi$ is the relative spectral phase between them, and t is the time delay. An example of a measured spectrum is presented in Supplementary Fig. S1b. After applying the Fourier transform, the modulated part can be distinguished in the side lobes (with separation proportional to pulse delay t). The relative spectral phase can be retrieved by the inverse Fourier Transform from one of either lobe signals. The example of a comparison between applied and retrieved 3rd-order spectral phase (φ_3=$3\cdot10^6\mathrm{fs}^3$) is presented in Supplementary Fig. S1c, showing excellent agreement within the pulse spectrum.

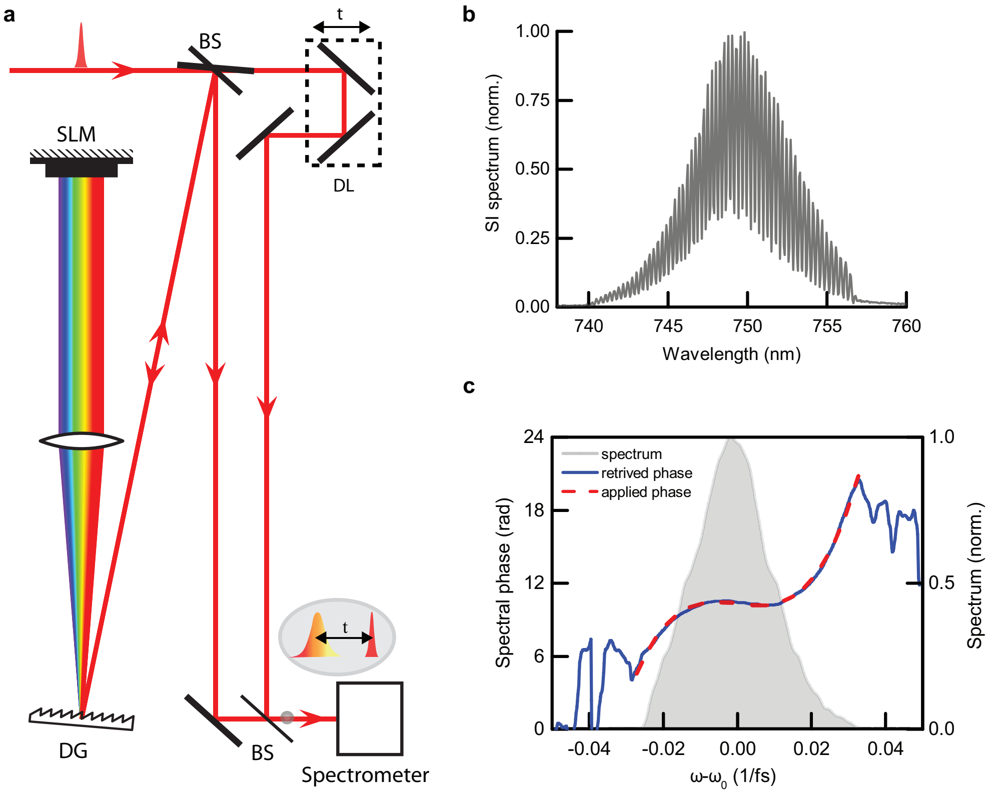

Supplementary Figure S1. Characterization of the pulse shaper by Fourier-transform spectral interferometry. (a) Schematic drawing of Mach-Zehnder interferometer with two copies of an ultrashort pulse created by a 50:50 low-dispersion non-polarizing beamsplitter (BS). One replica is shaped by the spatial light modulator (SLM) in the Fourier plane created by a diffraction grating (DG) and a long focal length lens (f=400 mm). The other pulse passes an optical delay line (DL) which controls the relative timing between two pulses t. The two pulses are combined by a second BS, and the modulated spectra are registered on the spectrometer. **(b)** Example of registered spectral interferometry (SI) data showing strong signal modulation (φ_3=$3\cdot10^6\mathrm{fs}^3$). **(c)** Retrieved spectral phase of the pulse from spectrum presented in (b). The retrieved phase shows perfect agreement with the applied phase on the SLM (φ_3=$3\cdot10^6\mathrm{fs}^3$, red dashed line).

Characterization of the STED pulses in the focal plane

The high-numerical-aperture objective lens introduces an additional spectral phase which smears out the temporal profile of an ultrashort pulse in the focal plane. We corrected this influence by an iterative procedure, monitoring two-photon excitation fluorescence (2PEF) in Coumarin 120 (Lambda Physik) dissolved in TDE. We iteratively maximized the 2PEF signal by changing the spectral phase on the shaper up to the 4th order. The final correction phase is presented in Supplementary Fig. S2a. Next, we measured the autocorrelation function (ACF) in the focal plane in the same Coumarin 120 solution, before and after the objective phase correction (Supplementary Fig. S2b). For corrected spectral phase, we observed higher signal and a narrower width of the ACF. A slightly asymmetrical ACF for the uncorrected pulse is related to spatial misalignment between the two pulses. The ACF width indicated a pulse duration of ∼130 fs in the focal plane, corresponding perfectly to the measured transform-limited pulse ACF at the back aperture by a commercial autocorrelator (data not shown). To explore, we also applied more complicated spectral phases to the STED pulse, to generate, from a single femtosecond pulse (∼130 fs), a burst of 9 pulses, each with ∼130 fs pulse duration separated by ∼500 fs (peak to peak). We calculated the necessary spectral phase for such an intensity profile by the iterative Fast Fourier Transform algorithm[2]. The spectral phase retrieved by SI and the expected temporal profile are presented in Supplementary Fig. S2c. To verify the temporal profile of the pulse in the focal plane, we measured the ACF as before, finding perfect agreement with the expected distribution (Supplementary Fig. S2d). The duration of the Gaussian STED pulse was controlled by applying different 2nd-order spectral phases (chirp), according to the relation:

$$\tau = \tau_0 \sqrt{1 + \left(4 \ln 2 \frac{\varphi_2}{{\tau_0}^2}\right)^2} \approx 4 \ln 2 \frac{\varphi_2}{\tau_0} \qquad (2)$$

where τ is the pulse duration, τ_0 is the transform-limited pulse duration and φ_2 is a 2nd-order spectral phase (i.e. pulse chirp). An up-chirped pulse corresponds to instantaneous frequency increases with time ($\varphi_2>0$), in down-chirped pulses frequency decreases with time ($\varphi_2<0$). We measured the STED pulse duration τ in the focal plane for different chirp values φ_2 (Supplementary Fig. S2e, Supplementary Table S1), finding a linear relation. In the short-pulse regime, the time delay between excitation and STED pulse was measured by the crosscorrelation function (CCF) of the ultrashort STED pulse replica with the excitation and STED pulse in the focal plane (in Coumarin 120 solution). The example of a CCF of the STED (750 nm) and excitation (635 nm) pulse is presented in Supplementary Fig. S2f. These measurements allow to precisely place the STED pulse after the excitation by use of the DL and estimate the excitation pulse duration to ∼500 fs in the focal plane.

Supplementary Table S1. Control of the STED pulse duration by the second-order spectral phase applied on the SLM. Autocorrelation function width (FWHM) as measured in the focal plane and deconvolved pulse duration (Gaussian, FWHM) for different 2nd order spectral phases (φ_2).

Spectral phase φ_2 (fs^2)	ACF width (ps)	pulse duration τ (ps)
0	0.179	0.127
$3 \cdot 10^4$	1.168	0.826
$6 \cdot 10^4$	2.276	1.626
$9 \cdot 10^4$	3.522	2.516

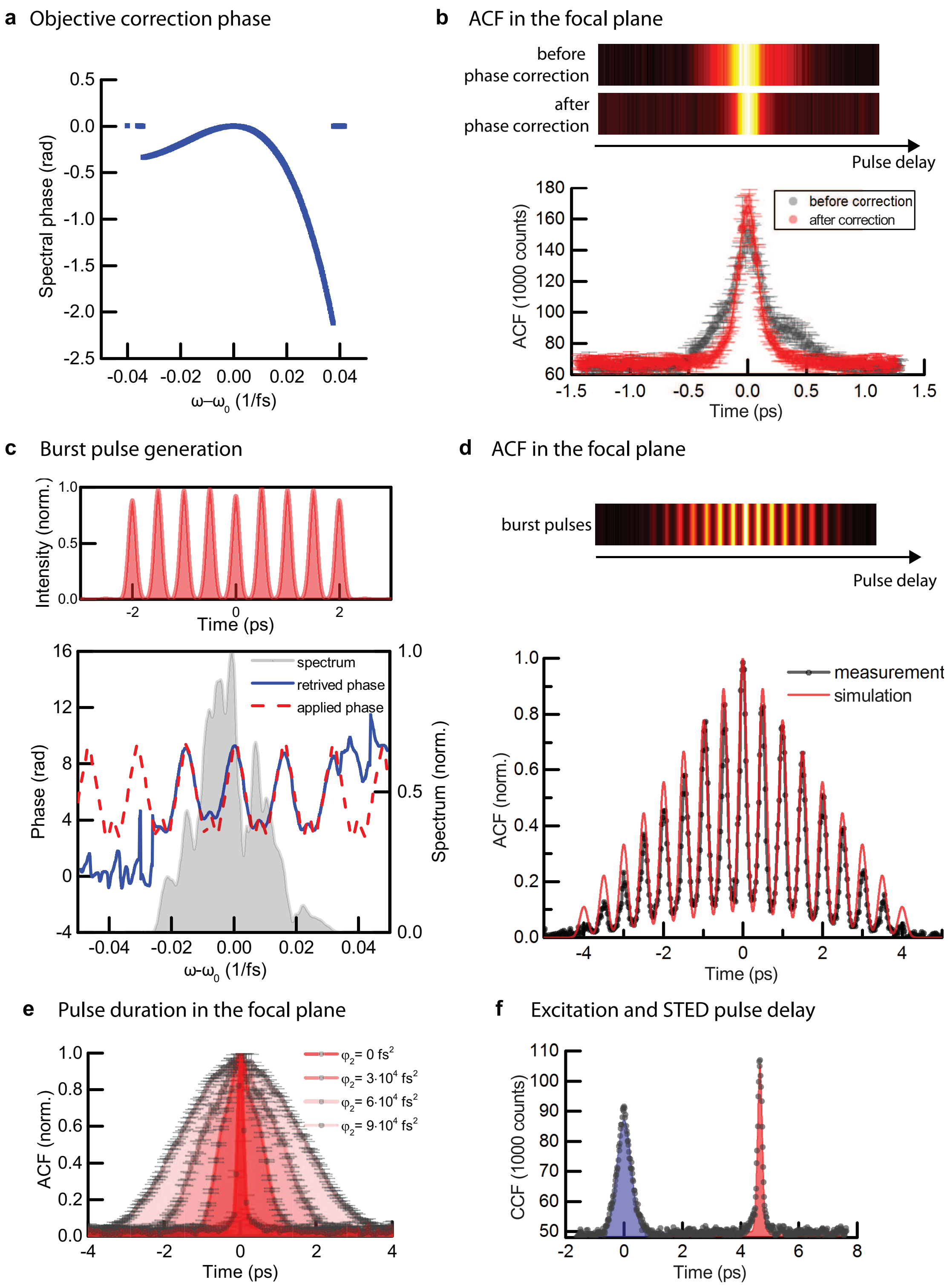

Supplementary Figure S2. STED pulse characterization in the ultrashort pulse regime (0.13−3 ps)**.** **(a)** Retrieved correction spectral phase for the objective lens. **(b)** Autocorrelation function (ACF) of an ultrashort pulse in the focal plane before and after the objective phase correction. The ACF was measured in Coumarin 120 solution by two-photon excitation fluorescence. **(c)** Generation of burst pulses from a single femtosecond pulse. Expected temporal intensity profile (top) and applied/retrieved spectral phase (bottom). **(d)** Measured ACF in the focal plane for burst pulses. The red line represents the expected ACF for burst pulses with the temporal profile shown in (c). **(e)** Control of the STED pulse duration in the focal plane by 2nd-order spectral phase φ_2. Intensity profiles represent the measured ACF in the focal plane. **(f)** Relative delay between excitation and STED pulse as measured by the crosscorrelation function (CCF) in the focal plane.

1.2 Long pulse duration regime ($10-500\,\mathrm{ps}$)

Characterization of STED pulse duration by autocorrelation

The pulse duration of long STED pulses was measured by a commercial autocorrelator (pulseCheck, APE) at the back aperture of the objective lens (Supplementary Fig. S3). The spectral phase introduced by the objective lens is negligible in this pulse duration regime. Before coupling the STED pulses to optical fibers, several high-dispersion glass rods were placed in the optical path to pre-stretch the ultrashort pulses and thus minimize nonlinear effects in the fibers. Different amounts of chirp were applied to the pulses by changing the length of polarization-maintaining fibers (Supplementary Table S2). The longest pulse duration ($\sim$500 ps) was at the edge of the autocorrelator measurement range. The time delay between excitation and STED pulse was controlled electronically to be approximately the FWHM of the STED pulse duration to maintain the highest possible de-excitation.

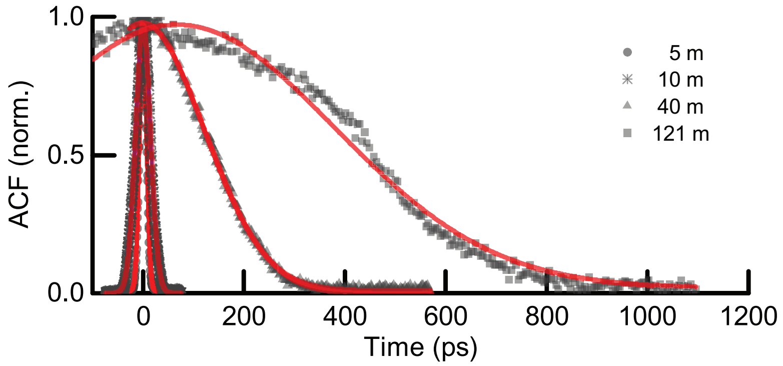

Supplementary Figure S3. STED pulse characterization in the long pulse regime ($10-500\,\mathrm{ps}$). Autocorrelation function (ACF), as measured at the back aperture of the objective lens for different lengths of the optical fibers. Red lines represent the Gaussian fits.

Supplementary Table S2. STED pulse duration with different optical fibers. Autocorrelation function width (ACF, FWHM), as measured at back aperture of the objective lens, and deconvolved STED pulse duration (Gaussian, FWHM) for different optical fibers. (*) Measurements for the longest pulses were at the edge of the autocorrelator range.

Fiber length (m)	Model, manufacturer	ACF width (ps)	pulse duration τ (ps)
5	PM630-HP, Thorlabs	16	11
10	PM630-HP, Thorlabs	34	25
40	PMC-600-4, Schäfter + Kirchhoff GmbH	283	200
121	PMJ-A3AHPC, OZ Optics	736	520*

2 Data analysis

An example of raw data from a measurement for a single pulse configuration is presented in Supplementary Fig. S4. First, we measured the reference fluorescence signal of the excitation pulse (Supplementary Fig. S4a). Then, the signal for the excitation and chopped STED beams acting together (Supplementary Fig. S4b) was measured and, finally, fluorescence induced by the chopped STED beam acting alone on the molecules (Supplementary Fig. S4c). Each measurement averaged signal over $\sim$100 s. The respective time traces are presented in Supplementary Fig. S4d. In the data analysis process, we normalized all curves to the initial fluorescence signal C_0 (excitation only) (Supplementary Fig. S4a). Then, we subtracted fluorescence induced by the STED beam C_S from the signal during exposure to both the excitation and STED beams together C_{ES} (Supplementary Fig. S4b minus Supplementary Fig. S4c). Next, we distinguished the fast fluorescence recovery component (de-excitation) from the slow recovery part (diffusion of fresh molecules, i.e., proportional to photobleaching) by an automated procedure incorporating the derivative of the second curve (Supplementary Fig. S4d, blue line). De-excitation, bleaching and STED-light-induced fluorescence were calculated as mean values from >10 recovery traces (Supplementary Fig. S4d). The error of measurement is estimated from the standard deviation of retrieved values. It is important to note that, for the same sample, often different offsets of photobleaching (low-order photobleaching) were observed due to aging, probably related to changes in oxygen concentration. For this reason, all measurements were performed in freshly prepared solutions. The relative dependence on the pulse duration (high-order photobleaching) was characterized by the same order of nonlinearity in all cases.

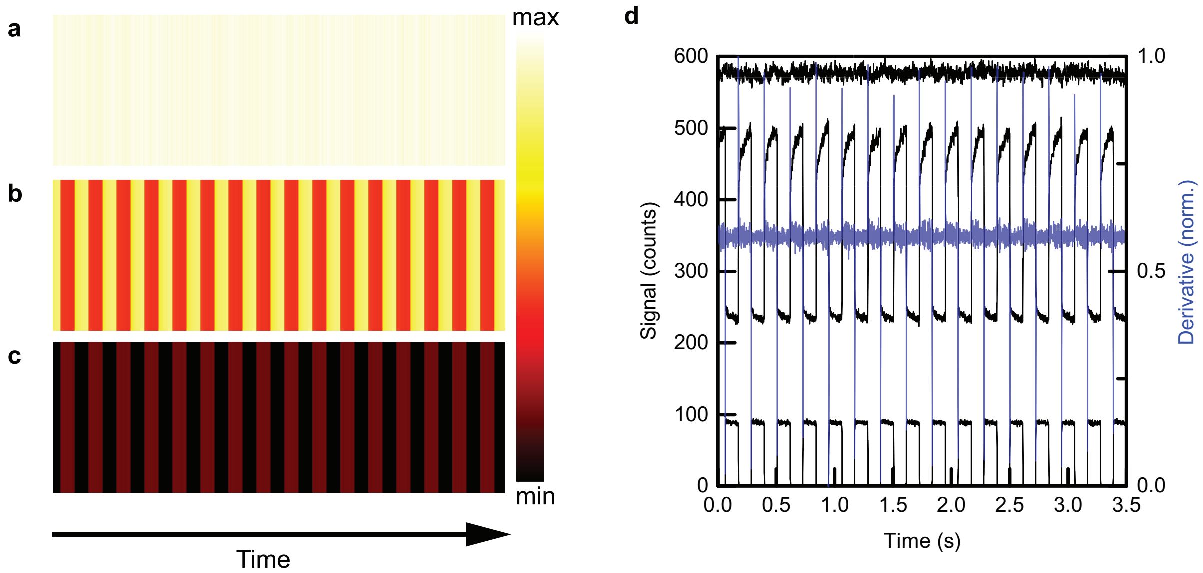

Supplementary Figure S4. Raw data (here: CW STED in ATTO647N TDE solution, 0.5 μM concentration, average over ~100 s.) **(a)** Fluorescence signal due to excitation pulses (635 nm, 30 µW average power). **(b)** Fluorescence recovery signal for excitation pulse (as previously) and chopped STED (750 nm, 150 mW average power). **(c)** Fluorescence signal induced by the chopped STED beam (as previously). **(d)** Intensity traces of (a-c). The blue line represents the derivative of (b).

3 Influence of the STED pulse chirp

We observed a noticeable dependence of bleaching and STED-light-induced fluorescence on the spectral chirp of the de-excitation pulse. For all tested dyes and peak intensities, down-chirped STED pulses showed ~25% lower STED-induced fluorescence and ~10% lower bleaching in comparison to commonly used up-chirped de-excitation pulses (Supplementary Fig. S5b,c). Within the measurement error, de-excitation was the same in both cases.

The influence of the chirp can be rationalized in the wave packet propagation picture (Supplementary Fig. S5a). Assuming two electronic potential surfaces V_0, V_1, the optical transition between them is possible only in case of resonance with an instantaneous optical frequency. After an ultrafast excitation, the wave packet propagates on an excited state potential (V_1) and quickly relaxes by vibrational relaxation (~1 ps) to the lowest energy level of V_1. Incoming de-excitation photons cause stimulated emission. The energy difference of the wave packet after de-excitation and excited state potential ($V_1 - V_0$) increases in time, and an up-chirped pulse follows this motion causing unwanted excitation by the STED light. A down-chirped pulse gives better results, as STED-light-mediated excitation is reduced, due to the opposite temporal distribution of spectral components within the pulse. This process will be more prominent for pulses with higher energy and was observed before in the context of efficient excitation in fluorescent dye solutions[3,4].

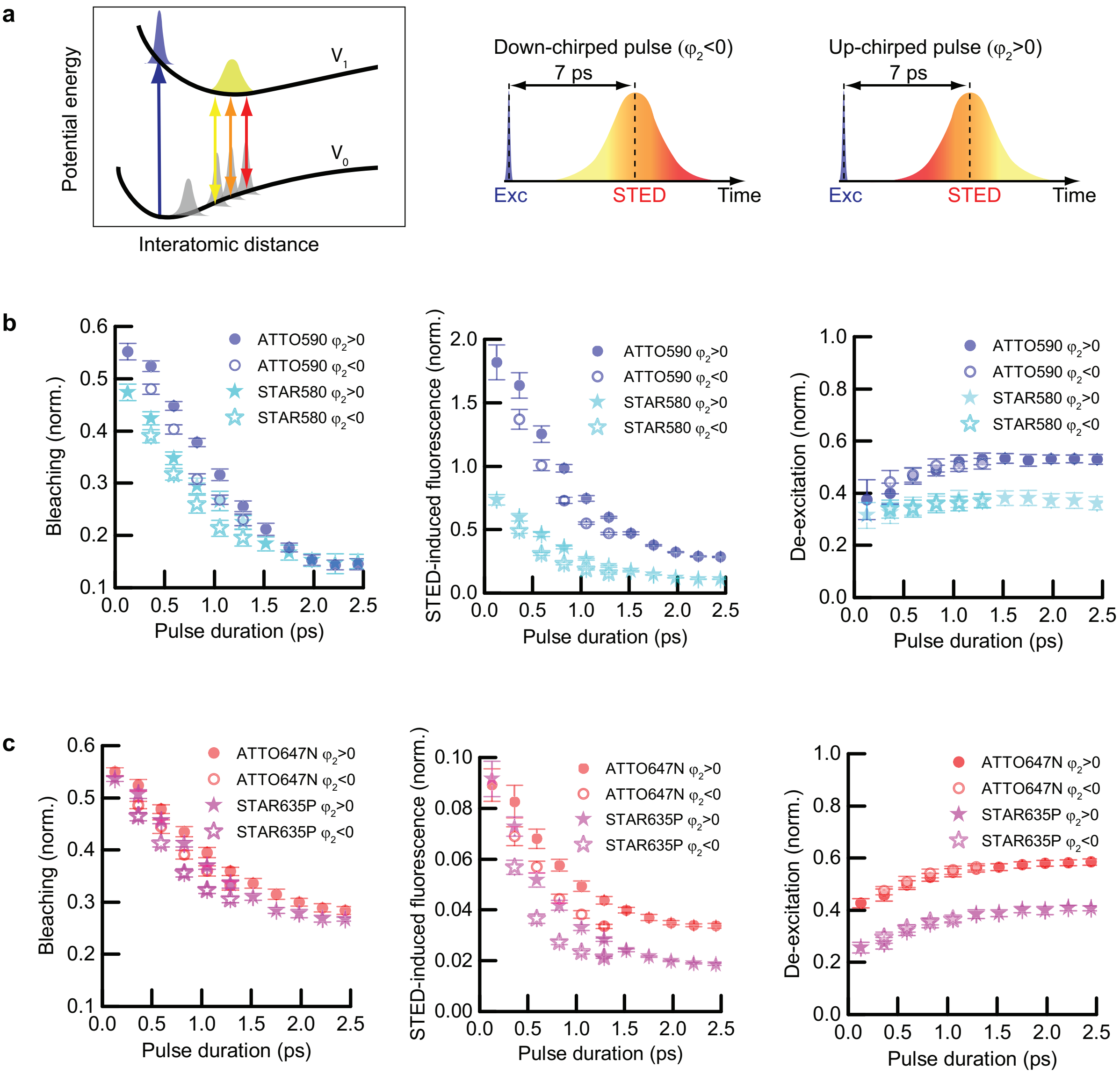

Supplementary Figure S5. Influence of the STED pulse chirp on the population of the excited state and photobleaching. (a) Schematic illustration of wave packet motion after de-excitation. The energy difference of the wave packet just after de-excitation and the excited-state potential ($V_1 - V_0$) increases in time, and an up-chirped pulse follows this motion, causing undesirable excitations of the just de-excited molecules. **(b)** Bleaching, STED-light-induced fluorescence and de-excitation as a function of pulse duration and chirp of the STED pulse for the dyes ATTO590 and STAR580. **(c)** Data as in (b) for the red dyes ATTO647N and STAR635P. All measurements were performed for an excitation power of 30 µW and a STED power of 10 mW (measured at the back aperture of the objective lens).

4 Simulation of de-excitation, bleaching and STED-light-induced fluorescence for ATTO647N

4.1 3D fitting procedure to experimental data

To model the behaviour of ATTO647N in STED microscopy for various STED photon fluxes we inferred the necessary probabilities of the involved processes (i.e. stimulated emission σ_{STED}, one/two-photon STED light absorption $\sigma_{1PE}/\sigma_{2PE}$ and photobleaching probability k) based on experimental data (Fig. 3a). We applied a 3D fitting procedure to include the 3D spatial profiles of the beams, and thus obtain more realistic parameters.
De-excitation (D), bleaching (B) and STED-light-induced fluorescence (SF) are defined as in the experiments:

$$D = 1 - \left(\frac{\sum_{x,y,z} \overline{S_1(t)}}{\sum_{x,y,z} \overline{S_1(t)}\big|_{I_{STED}=0}} - SF \right) \tag{3}$$

$$B = \frac{\sum_{x,y,z} \overline{\beta(t)}}{\sum_{x,y,z} \overline{S_1(t)}\big|_{I_{STED}=0}} \tag{4}$$

$$SF = \frac{\sum_{x,y,z} \overline{S_1(t)}\big|_{I_{Exc}=0}}{\sum_{x,y,z} \overline{S_1(t)}\big|_{I_{STED}=0}} \tag{5}$$

Temporal domain

The simplified model is presented in Supplementary Fig. S6. Most parameters are chosen as standard for fluorescent dyes and are fixed in the model (fluorescence lifetime τ_{fl}, vibrational relaxation γ_{vib}, excitation cross-section σ_{exc}, see Supplementary Table S3). The excitation pulse has a Gaussian shape with constant pulse duration τ_{exc}=500 fs and position t_0=1100 ps (Supplementary Fig. S6b). The STED pulse properties (duration τ, position t, average power P_{STED}) are varied according to experimental conditions. The numerical time axis has length t_{axis}=13 ns, which roughly corresponds to one cycle at the f=80 MHz repetition rate. We assumed that all events on this time scale will be repeating with the next pulses (steady-state approximation). The rate equations derived from the diagram of simplified molecular states (Supplementary Fig. S6a) are presented below:

$$\frac{\mathrm{d}}{\mathrm{d}t} S_1{}^* = (w_{exc} + w_{reexc})(S_0 - S_1{}^*) - \gamma_{vib} S_1{}^* - k(I_{STED}) S_1{}^* \tag{6}$$

$$\frac{\mathrm{d}}{\mathrm{d}t} S_1 = \gamma_{vib} S_1{}^* - \gamma_{fl} S_1 - w_{STED}(S_1 - S_0{}^*) - k(I_{STED}) S_1 \tag{7}$$

$$\frac{\mathrm{d}}{\mathrm{d}t} S_0{}^* = w_{STED}(S_1 - S_0{}^*) + \gamma_{fl} S_1 - \gamma_{vib} S_0{}^* \tag{8}$$

$$\frac{\mathrm{d}}{\mathrm{d}t} S_0 = \gamma_{vib} S_0{}^* - (w_{exc} + w_{reexc})(S_0 - S_1{}^*) \tag{9}$$

$$\frac{\mathrm{d}}{\mathrm{d}t} \beta = k(I_{STED}) \left(S_1 + S_1{}^*\right) \tag{10}$$

where $\gamma = \frac{1}{\tau}$, τ is the lifetime of a state; $w = \frac{\sigma I}{\hbar\omega}$, with σ a cross-section, I the light intensity, and $\hbar\omega$ the photon energy; $k(I_{STED})$ is the probability of photobleaching as a function of STED instantaneous intensity.

Assumptions and free parameters in the model:

(i) The population of each state is described by means of a probability, i.e. $\sum_i S_i = 1$.

(ii) Initially all molecules are in the ground state $S_0 = 1$.

(iii) Excitation of ground-state molecules by the STED beam (w_{reexc}) occurs by one- or two-photon absorption (σ_{1PE}, σ_{2PE}) and $w_{reexc} = \sigma_{1PE} \cdot \frac{I_{STED}}{\hbar\omega} + \frac{1}{2}\sigma_{2PE} \cdot \frac{I_{STED}}{\hbar\omega}^2$. The contributions of linear and two-photon absorption components have to be found experimentally. Note that in our simulation excitation by the STED beam occurs from the S_0 level, for simplicity. The more realistic situation for linear absorption at room temperature is presented in Fig. 5a. The interaction of molecules with the lower-energy STED-light photons leads to the population of higher vibrational levels of S_0, and is properly described by the Boltzmann distribution.

(iv) Bleaching by the excitation beam is not included in the model and negligible in comparison to STED-light-induced bleaching.

(v) Bleaching occurs only from the excited states (S_1, $S_1{}^*$) and is a monotonic function of STED intensity described as $k(I_{STED}) = k_1 \cdot (\frac{I_{STED}}{1W/m^2})^b$, where b is the order of nonlinearity of photobleaching. For ATTO647N, we measured $b = 1.4$. The photobleaching amplitude k_1 has to be found experimentally.

(vi) The probability (cross-section) of stimulated emission σ_{STED} has to be found experimentally.

Spatial domain

Beam intensities in the foci are approximated by 3D Gaussian beams (Supplementary Fig. S6c):

$$I(r,z) = \frac{2P}{\pi w(z)^2} \exp\left(-\frac{2r^2}{w(z)^2}\right) \tag{11}$$

where P is the average power, $w(z){=}w_0\sqrt{1+\frac{z}{z_R}}$ is the beam radius at the axial position z, $w_0{=}\frac{FWHM}{\sqrt{2\ln(2)}}$ is the beam radius in the focal plane, and $z_R{=}\frac{\pi {w_0}^2}{\lambda}$ is the Rayleigh range. Beam sizes are chosen as in the experiment, that is, the region of excitation in the focal plane is diffraction-limited (FWHM=227 nm); the STED beam is enlarged (∼1.5 of diffraction-limited size at the STED wavelength, FWHM=402 nm). The confocal detection corresponds to ∼1 Airy disc (AD) at the excitation wavelength.

Extraction of free parameters from the experimental data

All parameters employed in the model are listed in Supplementary Table S3. Values of free parameters (σ_{1PE},σ_{2PE},σ_{STED}, k_1; highlighted in yellow) were found by scanning the parameter space and comparing simulated values to the experimental data (Fig. 3a). The normalized root mean square error (nRMSE) quantifies the quality of the global fit to the data. nRMSE was $\leq$10% for all three curves. The final modelled values of de-excitation, bleaching, and STED-light-induced fluorescence as a function of STED pulse duration τ are presented as solid lines in Fig. 3a.

Discussion of the inferred free parameter values:

(i) The deduced probability of stimulated emission ($\sigma_{STED}{=}4.8\times10^{-18}\,\mathrm{cm}^2$) is in good agreement with the expected stimulated emission cross-section for fluorescent dyes of order $10^{-17}\,\mathrm{cm}^2$.

(ii) The linear absorption cross section of STED photons ($\sigma_{1PE} = 3.5\times10^{-21}\,\mathrm{cm}^2$) corresponds to the expected value at room temperature at the STED wavelength. In these conditions, higher vibrational levels of S_0 are occupied, and some molecules interact with the STED photons. The occupancy of higher vibrational states is described by the Boltzmann distribution, which well describes the red edge of the absorption band. Assuming that at an excitation wavelength $\lambda_{exc}{=}635\,\mathrm{nm}$ the absorption cross-section is $\sigma_{exc}(\lambda_{exc}){\approx}1\times10^{-16}\,\mathrm{cm}^2$, the one-photon absorption probability at the STED wavelength ($\lambda_{STED}{=}750\,\mathrm{nm}$) at room temperature ($T{=}293\,\mathrm{K}$) can be estimated as:

$$\sigma_{1PE} \approx \sigma_{exc}\cdot\exp\left(-\frac{E(\lambda_{exc})-E(\lambda_{STED})}{kT}\right) \approx 1.7\cdot10^{-5}\sigma_{exc} = 1.7\times10^{-21}\,\mathrm{cm}^2. \tag{12}$$

(iii) The two-photon excitation cross-section $\sigma_{2PE}{=}3.5GM$ roughly agrees with values measured for ATTO647N Streptavidin in phosphate buffered saline solution ($\sigma_{2PE}{=}8\pm4\,\mathrm{GM}$ at 750 nm)[5].

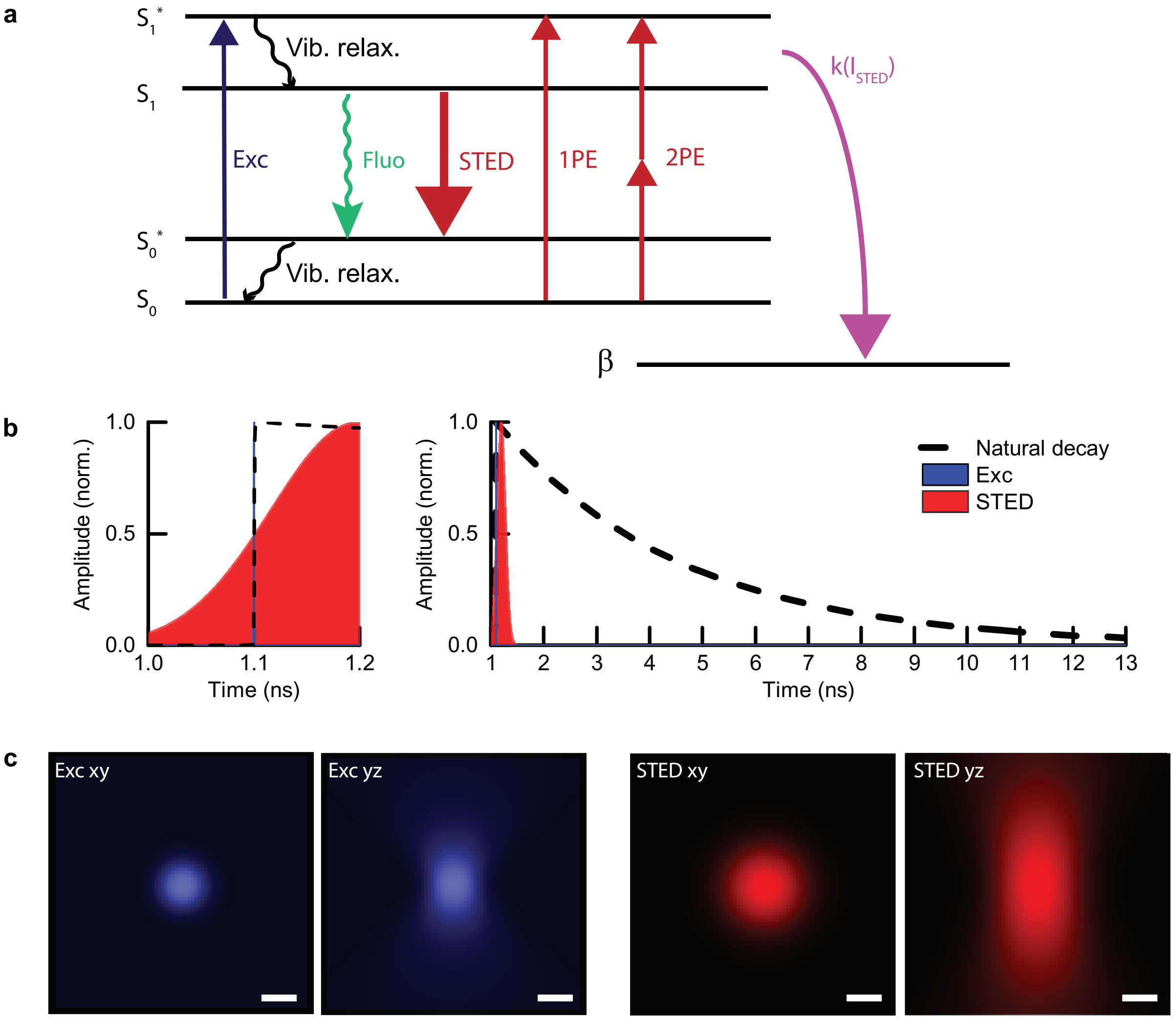

Supplementary Figure S6. Numerical simulation of de-excitation, bleaching and STED-light-induced fluorescence. (a) Simplified model of photobleaching in STED microscopy. **(b)** Temporal domain. Numerical time axis with excitation (blue) and STED (red) pulse profiles. The dashed line represents the natural fluorescence decay (i.e. STED power equals zero). **(c)** Spatial beam profiles for lateral (*xy*) and axial (*yz*) coordinates. All scale bars: 200 nm.

Supplementary Table S3. Parameters employed for modelling ATTO647N behaviour in STED nanoscopy. Fixed parameters are shown in white. Parameters extracted from fits to experimental data are highlighted in yellow. nRMSE represents a normalized root mean square error of the fit. The modelled curves and experimental data are presented in Fig. 3.

**Temporal domain**			
Parameter	Symbol	Value/Range	nRMSE
time step	dt	125 fs	
time range	t_{axis}	13 ns	
vibrational relaxation	t_{vib}	1 ps	
fluorescence lifetime	τ_{fl}	3.5 ns	
excitation cross-section	σ_{exc}	1×10^{-16} cm^2	
excitation pulse duration	τ_{exc}	500 fs	
excitation pulse position	t_{exc}	1.1 ns	
excitation power	P_{exc}	30 µW	
stimulated emission cross-section	σ_{STED}	4.8×10^{-18} cm^2	2%
STED pulse duration	τ	0.5 – 3000 ps	
STED pulse position	t_{STED}	1.1 ns+0.75τ	
STED power	P_{STED}	1 – 1000 mW	
STED one-photon excitation	σ_{1PE}	3.5×10^{-21} cm^2	10%
STED two-photon excitation	σ_{2PE}	3.5 GM	
photobleaching rate	k_1 (b=1.4)	5.2×10^{-10} Hz	5%
repetition rate	f	80 MHz	
**Spatial domain**			
Parameter	Symbol	Value/Range	nRMSE
radial step	dr	20 nm	
radial range	r_{range}	±200 nm	
axial step	dz	20 nm	
axial range	z_{range}	±700 nm	
excitation wavelength	λ_{exc}	635 nm	
STED wavelength	λ_{STED}	750 nm	
Factor of STED-focus enlargement	M	1.5	
Numerical aperture	NA	1.4	
Confocal detection	conf	1 AD	

4.2 2D STED microscope simulation

The 3D fitting procedure described above is costly in terms of time. In many practical cases, one can assume that the microscopic sample is two-dimensional (object is significantly thinner than the axial resolution, e.g. nuclear pore complexes). Calculations of resolution and spatial distribution of photobleaching were therefore performed for 2D intensity distributions, ignoring the axial dimension of the implemented profiles (Fig. 5, Supplementary Fig. S7). The radial step dr for these numerical simulations was decreased from 20 nm to typically 2 nm. In the focal plane, the excitation beam is approximated by a Gaussian function, and STED is represented by a first-order Laguerre-Gaussian beam (Fig. 5b top):

$$I_{exc}(r) = I_0 \exp\left(-\frac{2r^2}{w_0{}^2}\right) \tag{13}$$

$$I_{STED}(r) = I_0\left(\frac{2r^2}{w_0{}^2} + \zeta\right)\exp\left(1 - \frac{2r^2}{w_0{}^2}\right) \tag{14}$$

where w_0 is the beam width (excitation w_0=191 nm corresponding to FWHM=225 nm, STED w_0=285 nm and FWHM=190 nm /660 nm for inner/outer doughnut diameter respectively), ζ is the residual relative STED intensity at the targeted coordinate (in the minimum of the doughnut beam). Confocal detection is again defined as 1 AD at the excitation wavelength, corresponding to the final confocal diffraction-limited lateral resolution $\Delta r_{\mathrm{conf}}=\frac{\Delta r}{\sqrt{2}}\approx 162$ nm. Most parameters are fixed during simulation. The STED pulse properties are varied (duration τ, delay t, power P_{STED}). After applying the beams' spatial distributions in the model, we obtained the fluorescence profile and probability of photobleaching as a function of lateral position (Fig. 5b bottom). The resolution is defined as the FWHM of the fluorescence profile. The photobleaching value represents an integral over the spatial distribution of photobleaching probability (without confocal detection).

4.3 Resolution scaling with STED power

We modelled the resolution scaling of a STED nanoscope as a function of STED average power P_{STED}. As shown previously[6], the resolution follows a square-root law:

$$\Delta r = \frac{\Delta r_{conf}}{\sqrt{1+P_{STED}/P_{Sat}}} \tag{15}$$

where Δr_{conf}=162 nm, P_{STED} is the average STED power, P_{Sat} is a characteristic power to guarantee off-switching. We fit the formula (with a single free parameter P_{Sat}) to the modelled resolution scaling, finding excellent agreement with the expected behaviour (Supplementary Fig. S7a, P_{Sat}=23 mW; STED pulse duration τ=200 ps, STED delay t=150 ps, residual relative STED intensity at doughnut minimum ζ=0).

4.4 Optimal delay between excitation and STED pulse

The temporal distribution of de-excitation photons after the ultrafast excitation has an influence on the image quality. To enable a fair comparison between different STED pulse durations τ, we chose a favourable time delay t between the excitation and the STED pulse for non-gated detection (Supplementary Fig. S7b, circles) and gated detection (Supplementary Fig. S7b, dashed lines, starting at $t_{gate}=t+\frac{1}{2}\tau$). Taking into account the fluorescence decay, the flux of STED photons should be the highest just after the excitation, when many molecules relax by spontaneous emission. However, a simple overlap of the excitation and STED Gaussian pulses' maxima results in loss of half of the de-excitation photons: they do not contribute to depopulation of the excited state and, moreover, may interact with molecules in unwanted ways, causing excitation by the STED-light photons and photobleaching. On the other hand, a significant delay between pulses causes a reduction of the de-excitation efficiency because the STED intensity (and thus stimulated emission probability) is not high enough at the beginning of the fluorescence decay to compete with the spontaneous relaxation. This results in the early fluorescence component in the registered superresolution signal, creating wings (a halo) in the effective PSF[7]. To characterize the optimal conditions, we modelled the resolution as a function of delay t between excitation and STED pulse for different STED pulse durations τ (Supplementary Fig. S7b). The resolution changes as a function of delay t are highest for a low average STED power (~few mW at 80 MHz repetition rate), as the resolution curve is very steep in this regime (Supplementary Fig. S7a). To highlight the changes, we modelled the resolution for rather low constant time-averaged STED power P_{STED}=10 mW. The optimal delay (also for higher STED powers) depends on the pulse duration and is in the range ($\frac{1}{2}$FWHM; FWHM) of the STED pulse duration τ. We therefore selected the delay $t=0.75\tau$ for all investigated Gaussian pulses. The best resolution can be obtained for the shortest pulses ($\tau\approx$10 ps). Applying much longer pulses (~1 ns) reduces the achievable resolution due to early fluorescence[7]. This can be mitigated by applying gating, with the gating time equal to approximately the STED pulse duration: $t_{gate}\approx\tau$. In this case, the resolution is nearly independent of the pulse duration. The downside is a reduced fluorescence signal.

4.5 Bleaching at different STED powers

Similarly to the experimental results, our simulations reveal a sublinear scaling of bleaching with average STED power for the experimentally investigated power range (Supplementary Fig. S7c, left). Occupancy of the excited state (which largely initializes all bleaching pathways) is quite efficiently counter-acted by the STED light. In this regime, photobleaching may even be reduced in comparison to confocal microscopy measurements, because efficient transfer of the excited molecules to the ground state prevents them from following one of the possible destructive pathways. However, at higher average powers, the dependence of photobleaching should exhibit a superlinear scaling (Supplementary Fig. S7c, right). The reason is that, starting at higher STED pulse energies, the one-photon absorption events of STED light become equally significant as the absorption of photons from the excitation pulse. Thus, photobleaching initiated by STED-light-induced excitation of fluorophores becomes relevant, resulting in an increased region of photobleaching and a higher order of photobleaching scaling. A high probability of stimulated emission, and the typically confocal detection scheme employed in measurements, render the importance of STED-beam excitation events typically largely invisible in the detected fluorescence signal. As an example, for ATTO647N, the STED-light-induced fluorescence is $<0.5\%$ relative to the signal by excitation, at the average STED power $P_{STED}=1000\,\mathrm{mW}$ ($\tau=1000\,\mathrm{ps}$). However, the majority of bleaching is initiated by linear absorption of STED-light photons, and a reduction of the linear absorption coefficient (σ_{1PE}) by half reduces bleaching nearly by half (data not shown). Since the effects of unwanted one-photon absorption depend on the pulse energy rather than the temporal distribution of photons, this limits the STED pulse energies that can be applied (and thus resolution) before the fluorescent marker is photobleached. To reduce photobleaching, it is necessary to minimize the one-photon absorption coefficient by shifting the STED wavelength to longer wavelengths or changing the fluorescent dye to ones with bluer absorption spectra. For example, shifting the wavelength from 750 nm to 770 nm for ATTO647N should reduce the linear absorption cross-section approximately by an order of magnitude ($\sim$ten times), while the stimulated emission cross-section is reduced only by half.

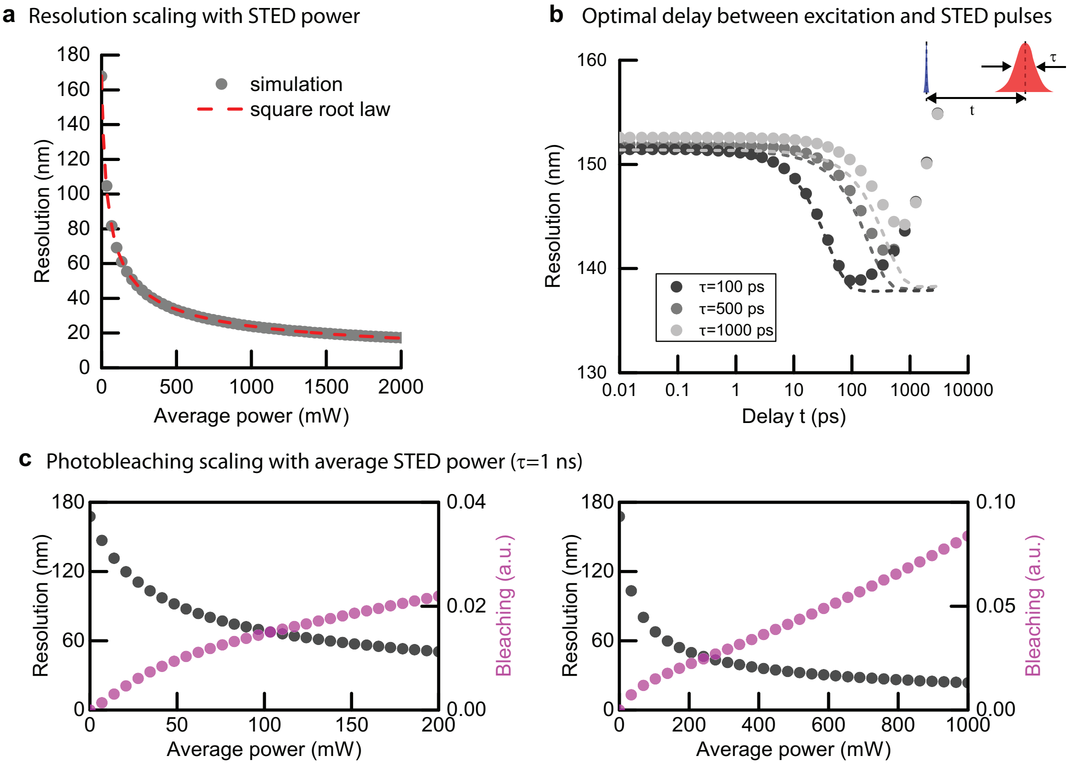

Supplementary Figure S7. Simulation of resolution and photobleaching for ATTO647N. (a) Resolution scaling with average STED power for STED pulse duration $\tau=200\,\mathrm{ps}$. The dashed line represents the square-root function with a single free parameter ($P_{Sat}=23\,\mathrm{mW}$). **(b)** Optimal time delay t between ultrafast excitation and STED pulse for different STED pulse durations τ ($P_{STED}=10\,\mathrm{mW}$). The dashed lines corespond to gated detection, with $t_{gate}=t+\frac{1}{2}\tau$. **(c)** Photobleaching and resolution vs. time-averaged STED power for moderate de-excitation powers (left, negligible linear absorption at STED wavelength) and higher powers (right). STED pulse duration $\tau=1\,\mathrm{ns}$, repetition rate $f=80\,\mathrm{MHz}$.

5 Simulation of de-excitation, bleaching and STED-light-induced fluorescence for ATTO590

The presented photobleaching model gives a good description if photobleaching follows a single intensity scaling. For the majority of the dyes (ATTO590, STAR580, STAR635P) the bleaching mechanism is more complicated (see Fig. 3b), indicating different dominant mechanisms for different peak intensity ranges. As, in general, photobleaching is associated with singlet and triplet ladders of states, the details of which were not accessible in our experiments, it was not possible to include all relevant parameters in the numerical modelling. A first obstacle is the much longer time scales for triplet-related processes (with lifetime of the first excited triplet state micro- to milliseconds) compared to singlet processes (with nanosecond lifetime of the first excited singlet state). A second obstacle lies in the significant increases in the numbers of free parameters, which are typically not directly measurable and often depend on the environment of the dye (such as ISC rate(s), triplet state lifetimes and associated photobleaching rates). Nonetheless, we modelled the behaviour of ATTO590 in STED microscopy, following the same procedure as described above (Section 4). Since the photobleaching differs for different STED intensity regimes, the photobleaching rate from the excited state S_1 in our model must reflect two of these regimes and can be approximated by:

$$k(I_{STED}) = k_0 \cdot (\frac{I_{STED}}{1W/m^2}) + k_1 \cdot (\frac{I_{STED}}{1W/m^2})^{1.9}. \quad (16)$$

The orders of nonlinearity were extracted from experimental data (see Fig. 3b, Table 1). The bleaching rates k_0, k_1 as well as the probabilities of stimulated emission σ_{STED} and STED light absorption by one- and two-photon processes σ_{1PE}, σ_{2PE} were found by fitting the model curves to the experimental data (see Supplementary Fig. S8a). All varied parameters are listed in Supplementary Table S4. As estimated based on normalized spectra, the stimulated emission probability is slightly lower than for ATTO647N, since the applied STED wavelength is further from the emission maximum of the dye (Fig. 1b). The probability of single-photon absorption is also lower since the STED wavelength is shifted from the absorption maximum. At the same time, ATTO590 is characterized by a significantly higher two-photon absorption probability. The experimental data and modelled curves are presented in Supplementary Fig. S8a,b). The presented simple numerical modelling only roughly describes the performance of ATTO590 and other markers in STED microscopy, as seen also from moderate but noticeable deviations in the power dependence of bleaching already for STED powers on the order of 100 mW in Supplementary Fig. S8b. This advises caution in the extrapolation to higher average STED powers and quantitative predictions of performance in the highest-resolution regime. The resolution and bleaching scaling for different STED pulse durations are presented in Supplementary Fig. S8c,d. ATTO590 shows slightly lower resolution than ATTO647N for the same average power. The bleaching has a stronger nonlinear component, however this difference manifests only at high STED peak powers (short pulses, compare Fig. 5c). With increasing STED pulse duration, the bleaching decreases to similar values as for ATTO647N. The resolution and bleaching scalings with STED time-averaged power are presented in Supplementary Fig. S8e,f. The STED pulse duration for the modelling equalled τ=1000 ps. The higher-order scaling of bleaching compared to ATTO647N suggests that further increases of pulse duration may indeed be beneficial for this dye. It is important to note that the spatial distribution of photobleaching is significantly different than for ATTO647N (compare Fig. 5f). Despite the different relative prominence of the involved optical transitions, the overall performance of the two dyes in STED microscopy is comparable.

Supplementary Table S4. Parameters extracted from experimental data for modelling ATTO590 behaviour in STED nanoscopy. All other parameters were fixed (see Supplementary Table S3). nRMSE is normalized root mean square error of the fit. The modelled curves and experimental data are presented in Supplementary Fig. S8a.

Temporal domain			
Parameter	Symbol	Value	nRMSE
stimulated emission cross-section	σ_{STED}	4×10^{-18} cm^2	4%
STED one-photon excitation	σ_{1PE}	8×10^{-22} cm^2	17%
STED two-photon excitation	σ_{2PE}	352 GM	
photobleaching rate	k_0 (b=1)	2.5×10^{-5} Hz	5%
photobleaching rate	k_1 (b=1.9)	9×10^{-18} Hz	

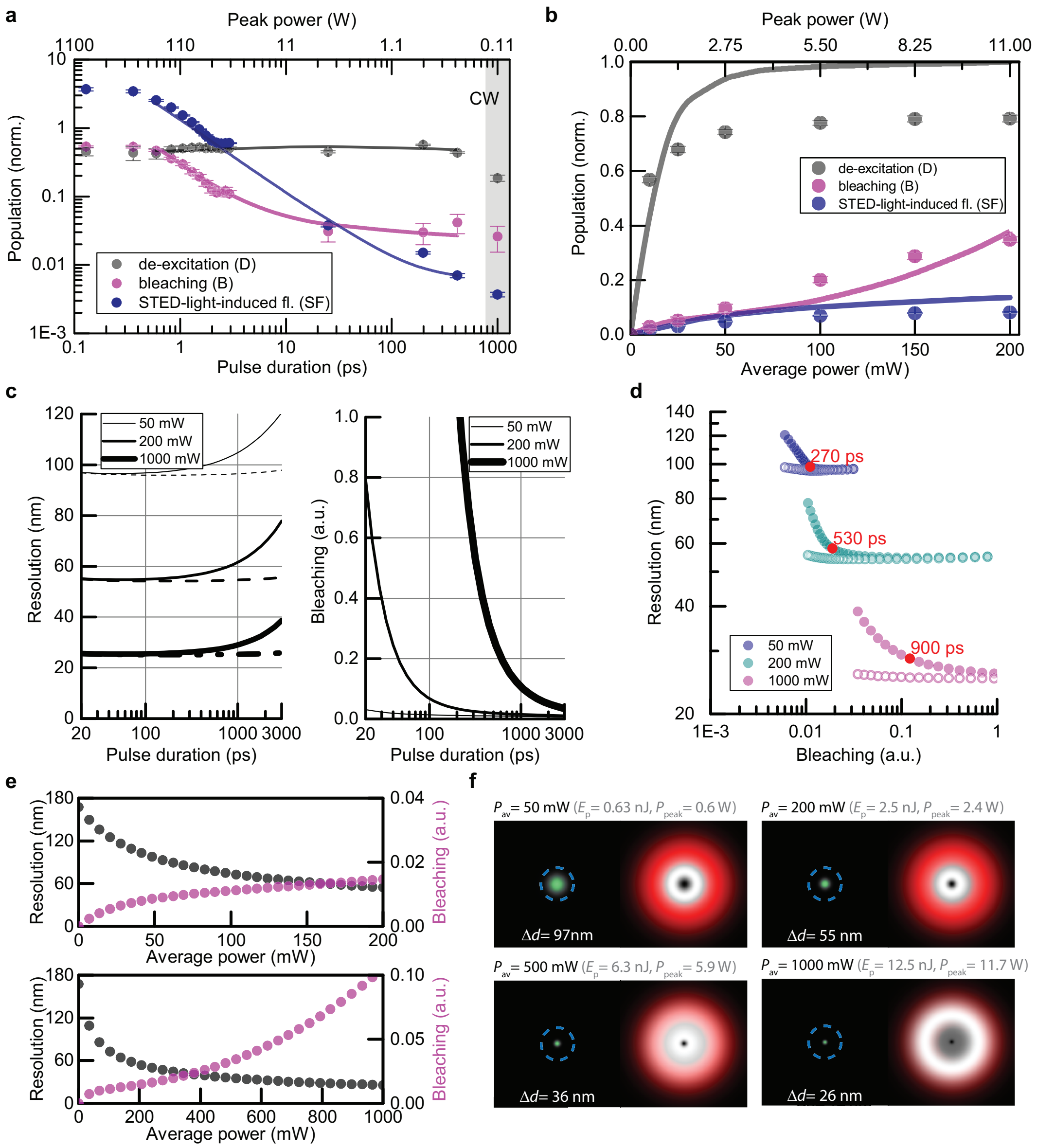

Supplementary Figure S8. Modelling ATTO590 in STED microscopy. **(a)** De-excitation, bleaching and STED-light-induced fluorescence vs. pulse duration for ATTO590. CW represents measurements for continuous-wave laser operation. The solid lines represent values calculated within the photobleaching model (see Supplementary Table S4). Time-averaged STED power: 10 mW (E_p=0.125 nJ) measured at the back aperture of the objective lens. **(b)** Comparison between experimental data and modelling for different time-averaged STED powers. STED pulse duration: τ=200 ps. **(c)** Resolution and bleaching as a function of pulse duration for three time-averaged STED powers. The dashed lines represent gated detection starting at $t_{gate} = \tau$. **(d)** The same data as in c presented as resolution vs. bleaching. The lowest bleaching and the highest resolution can be obtained by selecting the STED pulse duration and applying gated detection. **(e)** Photobleaching and resolution vs. time-averaged STED power for moderate de-excitation powers (top) and higher powers (bottom). STED pulse duration τ=1 ns. **(f)** Spatial distribution of fluorescence (green), normalized probabilities of photobleaching (linear grey scale, with highest photobleaching in white) and normalized STED beam profile as reference (red) for different average STED powers (STED pulse duration: τ=1000 ps). The stated resolution values Δd represent the respective FWHM of the fluorescence profile. The dashed blue circles correspond to the FWHM of the excitation focal spot.

Supplementary Table S5. Different implementations of STED microscopy with red dyes and fluorescent beads. *unpublished own measurements

#	Dye	λ_{STED} (nm)	τ (ns)	f (MHz)	P_{STED} (mW)	E_p (nJ)	$P_{STED}{}^{peak}$ (W)	Δr (nm)	Ref.
**1**	KK114	775	1	20	166	8.3	6.5	30	8
**2**	STAR635P	775	1.2	20	200	10	7.83	20	9
**3**	ATTO647N STAR635P	775	1	20	200	10	7.83	25	10
**4**	Crimson beads	750	0.2	80	300	3.75	17.6	40	*
**5**	ATTO647N	750	CW	–	423	5.3	0.42	50	11
**6**	ATTO633	750	0.2	76	114	1.5	7	40	12
**7**	ATTO633	735	0.09	1	2.4	2.4	25	40	13
**8**	fluorescent beads	763	CW	–	516	6.45	0.52	71	14
**9**	ATTO647N	770	0.25	80	168	2.1	7.89	85	15
**10**	Crimson beads	735	0.2	76	375	8.95	28	25	6
**11**	ATTO647N	760	0.2	76	70	0.921	4.33	89	16
**12**	ATTO647N	760	0.2	80	130	1.62	7.63	78	17
**13**	Crimson beads	730	CW	–	1250	15.625	1.25	34	11

6 Comparison: Resolution in STED microscopy reported for red dyes and fluorescent beads

The resolution improvement in STED microscopy relies on delivering to the sample a certain number of de-excitation photons, which force fluorophores to remain dark during signal detection in a spatially selective way. For de-excitation pulses shorter than the excited-state lifetime, the number of delivered photons (i.e., the pulse energy) roughly determines the resolution improvement (Supplementary Fig. S9a). For example, to obtain 25 nm resolution one needs to apply ~11 nJ pulses (back aperture), for both 1 ns and 200 ps STED pulses. These energies correspond to substantially different peak powers. In the first case, the peak power is ~7 W. Such peak powers were successfully implemented in STED microscopy before, for crimson beads as well as the fluorescent dye ATTO633 (e.g. points 4, 6, 7, 10). However, to deliver the same energy in 200 ps STED pulses would require a peak power of ~50 W, well above what has been reported so far (Supplementary Fig. S9b). Reducing the peak power by implementing long STED pulses directly diminishes the nonlinear component of photobleaching for fluorescent dyes.

It is important to note that the overall molecular photobleaching of fluorophores can be divided into two categories: low-order photobleaching and high-order photobleaching. In the first case, the photobleaching probability of the excited molecule is independent or linear with the applied light intensity. One possible pathway in this category is the photobleaching associated with the first triplet state. The magnitude of photobleaching in this case depends on the internal properties of the dye and its microenvironment (reactivity, intersystem crossing ISC) and the lifetime of the excited states (both singlet and triplet). For this bleaching mechanism (dominant in low-power applications, and especially important for blue-orange fluorescent dyes with high ISC), the optimal strategy is to reduce triplet build-up (by T-Rex, quenchers) and to push the excited molecules to the non-reactive ground state as soon as possible (short de-excitation pulses). In this case, application of STED pulses can even reduce photobleaching in confocal microscopy, due to a reduction of the effective time which molecules spend in the excited state. The other mechanism, high-order photobleaching, is associated with higher excited states (singlets and triplets) of the fluorophore, which can be reached by absorption of photons. As a result, the effective photobleaching rate from the excited state depends on the STED-light (photon flux) with order $b>1$. High-order photobleaching can be reduced by applying long de-excitation pulses up to approximately the excited-state lifetime of the fluorescent dye. Both processes contribute to the photodestruction of the fluorophore. For different markers and different environments, the major mechanism(s) may change, and further studies are necessary depending on the imaging context.

This study shows that, for ATTO647N, the scaling of photobleaching is nonlinear for all intensities investigated (b=1.4). This single bleaching scaling may be explained by its low ISC and thus low-order photobleaching, which is not limiting the performance of this dye in STED microscopy. For all other dyes, we report a nonlinear behaviour for the peak powers (>11 W) currently applied in STED microscopy to reach the best resolutions.

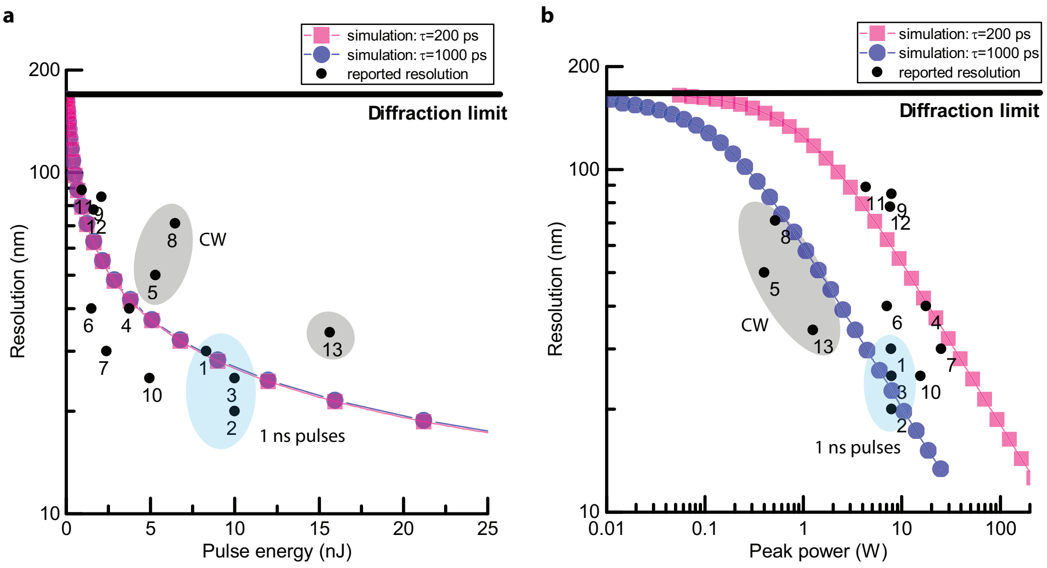

Supplementary Figure S9. Resolution reported for different STED implementations as a function of pulse energy (a) and pulse peak power (b) for different red dyes and crimson beads (see Supplementary Table S5). Pulse energies and peak powers were calculated at the back aperture of the objective lens. The data shown in colour represents simulation results for ATTO647N, for 200 ps (squares) and 1 ns (circles) Gaussian STED pulses at 80 MHz repetition rate. The top black line indicates the diffraction-limited confocal resolution for an excitation wavelength of 635 nm.

Parameter calculations

Time-averaged power at back aperture of the objective lens

All time-averaged STED powers were estimated at the back aperture of the objective lens. Since some publications specified this value in the focal plane, the powers at the back aperture were calculated as:

$$P_{back} = T^{-1} P_{focal} \tag{17}$$

where P_{back} is the average power at the back aperture, P_{focal} is the power in the focal plane, and T relates both measurements (and includes the transmission of the objective lens). T was calculated based on publications which stated both values[9,10] to T=0.6.

Supplementary References

1. Wong, V. & Walmsley, I. A. Analysis of ultrashort pulse-shape measurement using linear interferometers. *Opt. Lett.* **19**, 287–289 (1994).

2. Hacker, M., Stobrawa, G. & Feurer, T. Iterative Fourier transform algorithm for phase-only pulse shaping. *Opt. Express* **9**, 191–199 (2001).

3. Cerullo, G., Bardeen, C., Wang, Q. & Shank, C. High-power femtosecond chirped pulse excitation of molecules in solution. *Chem. Phys. Lett.* **262**, 362 – 368 (1996).

4. Cao, J., Bardeen, C. J. & Wilson, K. R. Molecular " π pulse" for total inversion of electronic state population. *Phys. Rev. Lett.* **80**, 1406–1409 (1998).

5. Velasco, M. G. M., Allgeyer, E. S., Yuan, P., Grutzendler, J. & Bewersdorf, J. Absolute two-photon excitation spectra of red and far-red fluorescent probes. *Opt. Lett.* **40**, 4915–4918 (2015).

6. Harke, B. *et al.* Resolution scaling in STED microscopy. *Opt. Express* **16**, 4154–4162 (2008).

7. Moffitt, J. R., Osseforth, C. & Michaelis, J. Time-gating improves the spatial resolution of STED microscopy. *Opt. Express* **19**, 4242–4254 (2011).

8. Schmidt, R. *et al.* Mitochondrial cristae revealed with focused light. *Nano Lett.* **9**, 2508–2510 (2009).

9. Göttfert, F. *et al.* Coaligned dual-channel STED nanoscopy and molecular diffusion analysis at 20 nm resolution. *Biophys. J.* **105**, L01 – L03 (2013).

10. Wurm, C. A. *et al.* Novel red fluorophores with superior performance in STED microscopy. *Opt. Nanoscopy* **1**, 7 (2012).

11. Willig, K. I., Harke, B., Medda, R. & Hell, S. W. STED microscopy with continuous wave beams. *Nat. Methods* **4**, 915–918 (2007).

12. Punge, A. *et al.* 3D reconstruction of high-resolution STED microscope images. *Microsc. Res. Tech.* **71**, 644–650 (2008).

13. Wildanger, D., Rittweger, E., Kastrup, L. & Hell, S. W. STED microscopy with a supercontinuum laser source. *Opt. Express* **16**, 9614–9621 (2008).

14. Liu, Y. *et al.* Achieving $\lambda/10$ resolution CW STED nanoscopy with a Ti:Sapphire oscillator. *PLoS ONE* **7**, e40003 (2012).

15. Bianchini, P., Harke, B., Galiani, S., Vicidomini, G. & Diaspro, A. Single-wavelength two-photon excitation-stimulated emission depletion (SW2PE-STED) superresolution imaging. *Proc. Natl. Acad. Sci. USA* **109**, 6390–6393 (2012).

16. Vicidomini, G. *et al.* STED nanoscopy with time-gated detection: Theoretical and experimental aspects. *PLoS ONE* **8**, e54421 (2013).

17. Pellett, P. A. *et al.* Two-color STED microscopy in living cells. *Biomed. Opt. Express* **2**, 2364 (2011).
